# Chiral Recognition Mechanism of Benzyltetrahydroisoquinoline Alkaloids: Cyclodextrin-Mediated Capillary Electrophoresis, Chiral HPLC, and NMR Spectroscopy Study

**DOI:** 10.3390/molecules30051125

**Published:** 2025-02-28

**Authors:** Erzsébet Várnagy, Gergő Tóth, Sándor Hosztafi, Máté Dobó, Ida Fejős, Szabolcs Béni

**Affiliations:** 1 Department of Pharmacognosy, Semmelweis University, Üllői út 26, H-1085 Budapest, Hungary; varnagy.erzsebet@phd.semmelweis.hu; 2 Center for Pharmacology and Drug Research & Development, Semmelweis University, Üllői út 26, H-1085 Budapest, Hungary; toth.gergo@semmelweis.hu (G.T.); hosztafi.sandor@semmelweis.hu (S.H.); dobo.mate@stud.semmelweis.hu (M.D.); 3 Department of Pharmaceutical Chemistry, Semmelweis University, Hőgyes Endre utca 9, H-1092 Budapest, Hungary; 4 Integrative Health and Environmental Analysis Research Laboratory, Department of Analytical Chemistry, Institute of Chemistry, ELTE Eötvös Loránd University, Pázmány Péter sétány 1/A, H-1117 Budapest, Hungary

**Keywords:** inclusion complexation, enantioseparation, enantiodiscrimination, laudanosine, tetrahydropapaverine, norlaudanosine, enantiomer migration order, sugammadex, chiral selector, polysaccharide-type column

## Abstract

The tetrahydroisoquinoline skeleton is a pharmacologically significant core structure containing chiral centers, making enantiomeric separation crucial due to the potentially distinct biological effects of each enantiomer. In this study, laudanosine (*N*-methyl-tetrahydropapaverine) and its three derivatives (6′-bromo-laudanosine, norlaudanosine, and *N*-propyl-norlaudanosine) were synthesized and used as model compounds to investigate chiral recognition mechanisms. Screening over twenty cyclodextrins (CyDs) as chiral selectors in capillary electrophoresis (CE), we found anionic CyDs to be the most effective, with sulfated-γ-CyD (S-γ-CyD) achieving a maximum *R_s_* of 10.5 for laudanosine. Notably, octakis-(6-deoxy-6-(2-carboxyethyl)-thio)-γ-CyD (sugammadex, SGX), heptakis-(2,3-*O*-diacetyl-6-*O*-sulfo)-β-CD (HDAS), heptakis-(2,3-*O*-dimethyl-6-*O*-sulfo)-β-CD (HDMS), and octakis-(2,3-*O*-dimethyl-6-*O*-sulfo)-γ-CD (ODMS) provided excellent enantioseparation for all four analytes. Following HPLC screening on CyD-based and polysaccharide-based chiral stationary phases, semi-preparative HPLC methods using amylose and cellulose-based columns were optimized to isolate enantiomers. The purity of the isolated enantiomers was evaluated by HPLC, and their configurations were confirmed via circular dichroism spectroscopy. The isolated enantiomers allowed us to explore enantiomer migration order reversals in CE and enantiomer elution order reversal in HPLC. Further ^1^H and 2D ROESY NMR experiments provided atomic-level insights into enantioselective complex formation, confirming enantiomer differentiation by SGX and elucidating the inclusion complex structure, where the ring C immersion into the CyD cavity is prevalent.

## 1. Introduction

Isoquinoline alkaloids are renowned for their diverse and potent pharmacological activities. This class includes analgesic compounds such as morphine and codeine, as well as anti-infective agents like berberine, palmatine, and magnoflorine. Due to the wide range of biological activities, these alkaloids hold increasing significance in drug development, and with synthetic derivatives (e.g., drotaverine) serving as prominent pharmaceutical agents [1].

**Figure 1:**
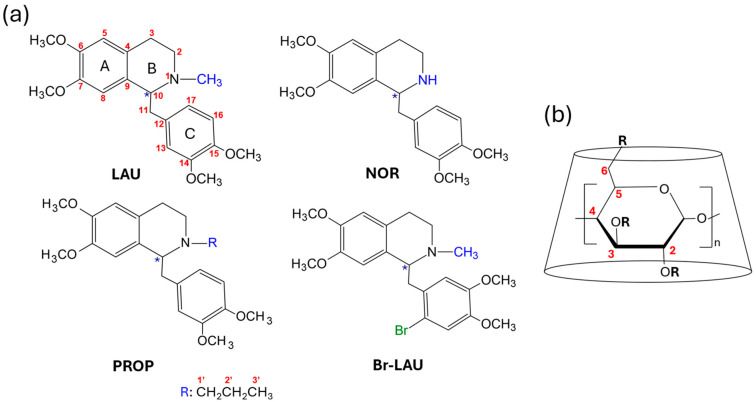
(**a**) The chemical structure of the studied racemic compounds: laudanosine (LAU), norlaudanosine (NOR), *N*-propyl-norlaudanosine (PROP), and 6′-bromo-laudanosine (Br-LAU). The chiral centers are marked with asterisks. (**b**) The schematic representation of cyclodextrins.

Beyond their pharmacological applications, isoquinoline alkaloids represent one of the largest structural classes of natural compounds, found across various plant families, including Papaveraceae, Berberidaceae, Fumariaceae, Menispermaceae, Ranunculaceae, Rutaceae, and Annonaceae [2]. These compounds are biosynthesized from phenylalanine or tyrosine and are characterized by an isoquinoline or tetrahydroisoquinoline core structure. The chirality of the skeleton is established early in the biosynthetic pathway, through the condensation of an appropriate amine and aldehyde, resulting in the formation of (*S*)-norcoclaurin [3]. In the biosynthesis of papaverine in *Papaver somniferum* L. (Opium poppy), tetrahydroisoquinoline structures are prevalent, and laudanosine (*N*-methyl-1,2,3,4-tetrahydropapaverine, LAU) is a degradation product of papaverine. (*R*)-LAU, derived from (*R*)-reticuline, has also been identified in small amounts in other *Papaver* species, such as *Papaver macrostomum*, where it was first isolated [4]. LAU and its *N*-demethylated form, norlaudanosine (1,2,3,4-tetrahydropapaverine, NOR) are also metabolites of neuromuscular blocking agents atracurium and cisatracurium, which are widely used in anesthesia [5]. Their synthetic analogues *N*-propyl-norlaudanosine (*N*-propyl-1,2,3,4-tetrahydropapaverine, PROP) [6] and 6′-bromo-laudanosine (Br-LAU) were studied for their potential anticancer properties in multidrug resistance (MDR) [7,8]. Similarly, Zeng et al. investigated the inhibitory effects of several tetrahydroisoquinoline-skeletal compounds on P-glycoprotein, concluding that this core holds potential as a viable scaffold for P-glycoprotein inhibitor drugs [9]. This also highlights the promise of benzyltetrahydroisoquinoline-based compounds as targets for novel cancer chemotherapeutics addressing MDR. For the chemical structure of the studied alkaloids, see Figure 1.

Several alkaloids with a tetrahydroisoquinoline skeleton, such as higenamine—widely used in traditional medicine and dietary supplements—exhibit pharmacological activity predominantly through their (*S*)-enantiomer [10,11]. Likewise, antidepressants with similar core structure, such as nomifensine and diclofensine, owe their activity to the (*S*)-enantiomer [12,13].

Given the chirality of the tetrahydroisoquinoline skeleton and the possible distinct biological effects of its enantiomers, comprehensive chiral separation studies could be essential. Capillary electrophoresis (CE) offers a high-throughput, cost-effective solution with minimal sample consumption and straightforward method optimization for this purpose. CE employs a variety of chiral selectors, including native and derivatized CyDs, crown ethers, and antibiotics, which can be used as buffer additives for chiral resolution, even as dual systems enhancing separation [14,15]. In contrast, high-performance liquid chromatography (HPLC) relies on chiral selectors immobilized on the column, providing a more robust and scalable platform, albeit with fewer selector variations and higher costs. HPLC is particularly advantageous for (semi-)preparative applications, especially when employing the polar organic mode, which simplifies method development and promotes efficient solvent evaporation [16]. CE and HPLC thus complement each other well.

CyDs are frequently used as chiral selectors in CE due to their ability to efficiently recognize and separate enantiomers. These cyclic oligosaccharides (see Figure 1b for their schematic structure) are composed of α-(1,4)-linked *D*-glucopyranose units. The three most prevalent CyDs—α-CyD, β-CyD, and γ-CyD—are composed of six, seven, and eight glucose units, respectively, forming truncated cone structures that vary in size. The inner cavity is relatively hydrophobic, which can accommodate apolar guest molecules via inclusion complex formation. While the outer surface is more hydrophilic, allowing CyDs to enhance the solubility of poorly water-soluble compounds. Through various synthetic modifications, CyDs can be fine-tuned for specific applications. For instance, methylation (e.g., randomly methylated γ-CyD, RAME-γ-CyD) increases hydrophobicity and can enhance selectivity for certain analytes [17], while hydroxypropylation e.g., hydroxypropyl-β-CyD (HP-β-CyD) improves the aqueous solubility of the β-CyD [18]. Sulfate groups e.g., sulfated-β-CyD (S-β-CyD) and sulfated-γ-CyD (S-γ-CyD) or carboxylate functions e.g., carboxymethyl-β-CyD (CM-β-CyD) and carboxymethyl-γ-CyD (CM-γ-CyD) introduce negative charges, enabling electrostatic interactions with cationic analytes and further improving separation efficiency [19]. The diverse cavity sizes and functional groups of CyDs enable tailored separations, enhancing enantioselectivity and resolution. Although previous studies have explored the enantiomer separation of LAU using CE with polysaccharides, chondroitin sulfate-glycogen dual systems, and polymerized chiral micelles as chiral selectors, neither comprehensive CE screening nor baseline separation has not yet been conducted [20,21,22,23,24,25]. Regarding the LAU analogues, only the enantiomer excess determination was conducted following the enantioselective synthesis of (*R*)-NOR using chiral HPLC on a Chiralcel OD polysaccharide column under normal-phased mode [26].

To understand the molecular mechanisms underlying CyD-mediated CE, complementary techniques such as nuclear magnetic resonance (NMR) spectroscopy and computational modeling are essential. NMR spectroscopy provides atomic-level insights into CyD-alkaloid complexes, elucidating their dynamic properties and fine structures under conditions comparable to CE enantioseparations. For this purpose, ^1^H NMR and 2D Rotating Frame Overhauser Enhancement Spectroscopy (ROESY) methods are suitable [27,28,29]. Molecular modeling has been applied for structurally related compounds such as salsolinol, *N*-methylsalsolinol, and 1-benzyltetrahydroisoquinoline, which share structural similarities with LAU [30].

To the best of our knowledge, no studies have yet attempted to separate LAU enantiomers using CyD-based chiral selectors or to investigate their CyD complexes via NMR spectroscopy. This study addresses these gaps by exploring the chiral recognition mechanism of LAU with CyDs. To gain deeper insights into the chiral interactions involved, three LAU derivatives were also synthesized and included in the chiral CE, HPLC, and NMR studies.

## 2. Results and Discussion

### 2.1. Synthetic Procedures

**Scheme 1:**
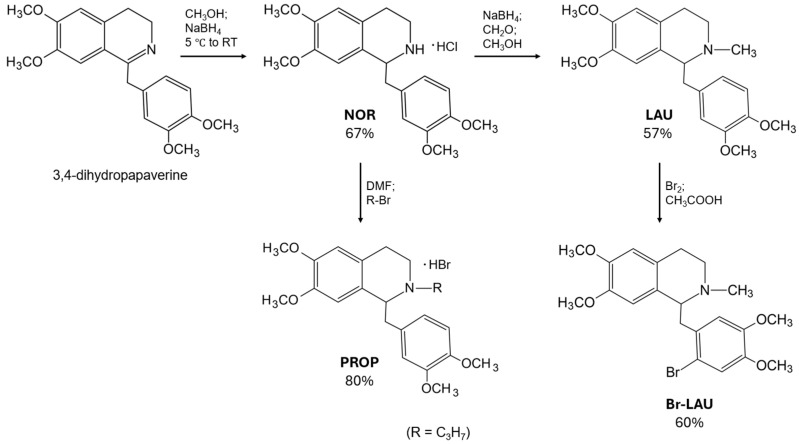
Synthesis of norlaudanosine and its derivatives: norlaudanosine (NOR), laudanosine (LAU), *N*-propyl-norlaudanosine (PROP), 6′-Br-laudanosine (Br-LAU). Further conditions can be found in Section 3.2.

Several methods have been reported in the literature for the synthesis of LAU and its derivatives [9,31,32,33,34,35]. In our study, we adopted a modified approach to improve solubility, reduce reaction time, and enhance the efficiency of key transformations. Briefly, 3,4-dihydropapaverine was used as the starting material. Its reduction with sodium borohydride in methanol (MeOH) yielded racemic NOR, based on the synthesis method of Kametani et al. [36]. This racemic benzyltetrahydroisoquinoline was further modified to obtain LAU via reductive amination [37,38,39]. The *N*-alkylation of NOR with propyl-bromide to obtain PROP was performed in dimethylformamide (DMF) at 60 °C for 20 h. This approach differs from the previously published 48 h reflux method, as DMF offers better solubility for NOR derivatives, leading to a more efficient alkylation process. Additionally, we prepared the hydrobromide salt instead of the previously reported hydrochloride salt, which proved to be more advantageous for our study. Notably, the previously described quaternization–*N*-demethylation method differs fundamentally from our direct *N*-alkylation strategy [40]. The synthesis of Br-LAU was achieved via direct bromination of LAU. In contrast, the previously published study [41] utilizes bromination of *N*-formyl-norlaudanosine, followed by formyl group reduction. Additionally, the racemic Br-LAU compound has been reported in earlier literature [42], where it was obtained via methylation of 6-Br-papaverine and subsequent reduction of the resulting quaternary salt. Further details are provided in Scheme 1 and Section 3.2.

### 2.2. Analytical and Semi-Preparative Chiral HPLC Studies

The enantioseparation performance, resolution and enantiomer elution order (EEO) of three polysaccharide-type and three CyD-based chiral columns were evaluated for the four synthesized racemic alkaloids. The CyD-based stationary phases included Shiseido Chiral CD-pH, which contained phenylcarbamate-β-CyD; Astec Cyclobond I 2000, incorporating native β-CyD and Nucleodex β-PM, containing permethylated β-CyD. The polysaccharide-based columns were: Chiralpak AD and Chiralpak AI, both containing amylose tris(3,5-dimethylphenylcarbamate) and Chiralcel OD, which contains cellulose tris(3,5-dimethylphenylcarbamate) [43]. The results are summarized in Table 1, and representative chromatograms are depicted in Supplementary Materials Figures S1–S4. It can be observed that polysaccharide columns are superior to CyD columns. Using native and permethylated β-CyD-based columns, no enantiorecognition was observed, while with the phenylcarbamate-β-CyD-based column, only NOR could be separated, albeit with a low-resolution value. In contrast, using the polysaccharide-type stationary phases, it was possible to enantioseparate all derivatives. The highest resolution value (*R_s_* > 2.5) was achieved for LAU on the Chiralcel OD column, independent of the applied mobile phase. Excellent enantioseparation of NOR enantiomers was achieved using both Chiralpak AD and Chiralpak IA columns, regardless of the mobile phase applied. Additionally, Chiralcel OD provided adequate separation (*R_s_* > 1.5) when using an acetonitrile (ACN) based mobile phase. For Br-LAU, the highest resolution was accomplished on Chiralcel OD with a MeOH-based mobile phase. For the propyl-derivative, the highest resolution value (*R_s_* = 1.9) was observed on Chiralpak AD with a MeOH-based mobile phase. Interestingly, Chiralpak AD outperformed Chiralpak IA in terms of enantioresolution when MeOH was used as the eluent, despite both columns utilizing the same chiral selector. The observed difference in performance can be attributed to the distinct immobilization methods of the chiral selector, with the coated phase of Chiralpak AD potentially offering greater flexibility in chiral recognition under these specific conditions.

These results provided a strong basis for the semi-preparative isolation of the individual enantiomers of the synthesized alkaloids. A semi-preparative Chiralcel OD column with MeOH as the eluent was chosen for isolating the enantiomers of LAU and Br-LAU, while a semi-preparative Chiralpak AD column with MeOH was utilized for the milligram-scale isolation of PROP enantiomers. Since the semi-preparative columns differed from their analytical counterparts only in column diameter (10 mm vs. 4.6 mm), no additional method development was required. The recovery was approximately 90% and the enantiomeric purity of one Br-LAU enantiomer was 91%, while all other compounds achieved purities exceeding 95%. These results underscore the advantages of the polar organic mode for semi-preparative applications.

**Figure 2:**
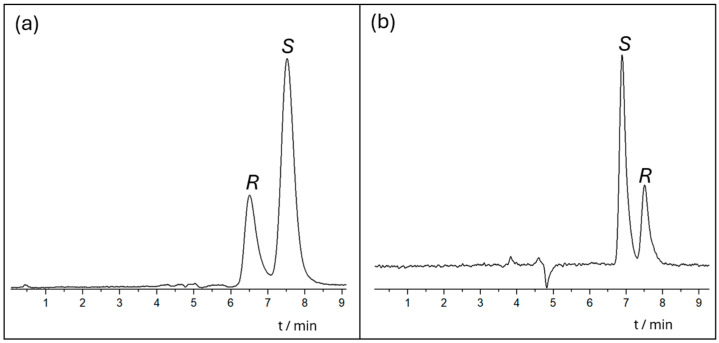
HPLC chromatograms depicting the enantiomer elution order reversal for PROP applying (**a**) Chiralpak AD and (**b**) Chiralcel OD column, using the same eluent: MeOH modified with 0.1% diethylamine (DEA). Racemic samples were spiked with (*S*)-PROP. Further conditions can be found in Section 3.3. Chiral HPLC.

To determine the configuration of the isolated enantiomers, circular dichroism spectroscopy (CD) was utilized using (*S*)-NOR as a reference compound [44]. Representative CD spectra are provided in Supplementary Figures S5–S8. Following stereochemical analysis, the EEO on polysaccharide columns was also determined and is summarized in Table 1. The results indicate that the EEO is predominantly *S*,*R* under most tested conditions; however, an *R*,*S* order was exclusively observed for PROP on both Chiralpak columns (see Figure 2).

### 2.3. Enantioseparation of the Benzyltetrahydroisoquinoline Alkaloids by CE

The investigated benzyltetrahydroisoquinoline alkaloids are basic compounds bearing tertiary (LAU, Br-LAU, and PROP) or secondary amine (NOR) functions. Among them, the least basic compound is Br-LAU (with predicted p*K_a_* of 7.77), followed by LAU (8.05) and PROP (8.59), while the secondary amine derivative NOR is the most basic compound with a predicted p*K_a_* of 8.96. The aforementioned p*K_a_* values were predicted using the ChemAxon Online Platform [45]. As we aimed to include charged and non-charged CyDs in our CE study, the selection of an appropriate background electrolyte (BGE) was needed. Sugammadex (SGX) and its analogues exhibit pH-dependent water solubility, which is optimal in the neutral to slightly alkaline pH range (approximately pH 7–8). Under acidic conditions (pH < 5), their solubility decreases due to protonation of functional groups, while at highly alkaline pH (>9), structural changes might also decrease their solubility. A 30 mM phosphate buffer (pH 7.4) proved to be suitable for our study, resulting in symmetrical peak shapes and short analysis times; furthermore, this pH can be considered as a biorelevant condition.

Over twenty CyDs—including neutral and negatively charged derivatives, listed in Section 3.1. Materials—were screened to evaluate their chiral selector performance towards the racemic benzyltetrahydroisoquinolines. Chiral recognition was observed with several CyDs; see Table 2 for the chiral resolution values.

Native CyDs exhibited no enantiorecognition, while among the neutral CyDs, only the hydroxypropylated CyDs showed limited enantioselectivity. Among the various substituted CyDs tested, anionic CyDs proved to be the most effective, and the sulfation emerged as the most advantageous modification of the CyD rim in terms of enantioseparation of LAU derivatives. The highest resolution for LAU and for Br-LAU was obtained with the randomly sulfated S-γ-CyD (*R_s_* 10.5 and 7.3, respectively). NOR enantiomers were best resolved using the single isomer ODMS (*R_s_* 7.5), while the highest resolution for PROP enantiomers was achieved with its beta analogue HDMS (*R_s_* 7.2). Besides sulfated CyDs (e.g., the single-isomer HDAS, HDMS, and ODMS), SGX was found to be the most effective selector, providing baseline resolution for all four LAU derivatives (see Table 2 for *R_s_* values and Section 3.1. for CyD abbreviations).

Additionally, apparent averaged complex stability constants and complex mobilities were also determined for several complexes (see Supplementary Materials Tables S1 and S2). To the best of our knowledge, the stability constants of LAU and its derivatives with CyDs have not been previously reported. In this study, we found that the complex stability constant values are generally below 2000 M^−1^, while the highest complex stabilities were achieved using SGX: with (*S*)-LAU the complex stability constant was 2070, and with (*R*)-LAU the SGX complex reached 3590 M^−1^. These values align well with typical stability constants obtained for alkaloid-CyD complexes [27,46,47]. The enantiomeric discrimination process was mainly driven by the aforementioned different stability of the transient diastereomeric complexes. Contrary to that, with S-β-CyD or CM-γ-CyD, the chiral recognition is driven by different complex mobilities, while in the case of Br-LAU with SGX, the difference in complex mobility and complex stability constants values together are responsible for the chiral separation. For LAU, several CyDs were studied to establish structure–stability relationships (see Supplementary Materials Tables S1 and S2). While no definitive conclusions could be drawn regarding the effect of CyD cavity size, the charge of the selectors’ sidechain was found to significantly enhance the stability in all cases.

### 2.4. Enantiomer Migration Order Reversal Determination by CE

Accurate detection of enantiomeric impurities is critical for pharmaceutical quality control. CE has proven effective for quantifying enantiomeric impurities at levels as low as 0.05% [48]. However, when the eutomer concentration reaches several tens of milligrams per milliliter, the distomer peak at just a few micrograms per milliliter—several orders of magnitude smaller—can be obscured by the larger eutomer peak, particularly if the former exhibits tailing [49]. To mitigate this, it is essential for the distomer to migrate before the eutomer, ensuring a suitable baseline resolution.

**Figure 3:**
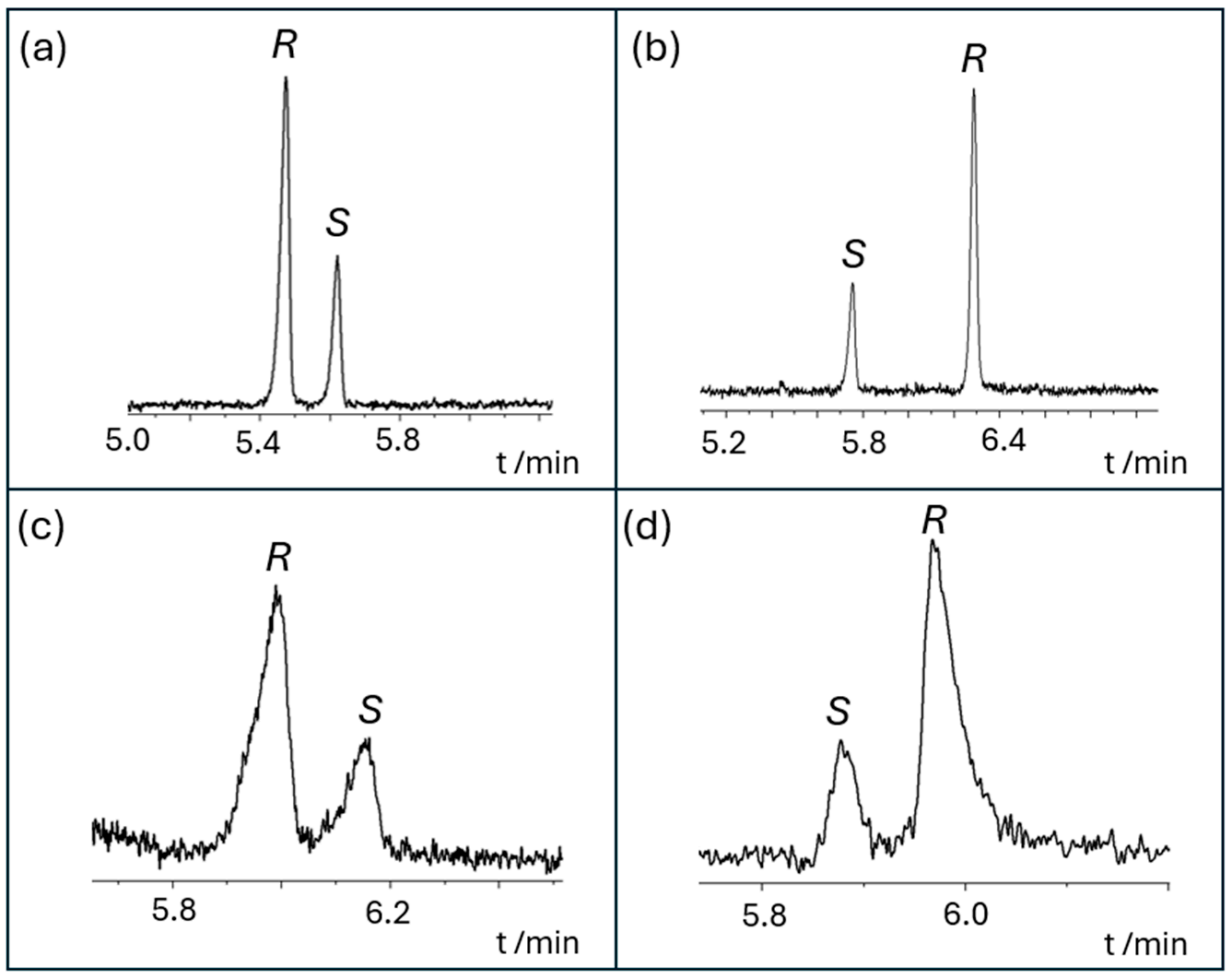
Partial electropherograms depicting the cavity size-dependent EMO reversal for LAU applying (**a**) S-β-CyD, and (**b**) S-γ-CyD, and Br-LAU using (**c**) SBX and (**d**) SGX, as chiral selector. Racemic samples were spiked with the (*R*)-enantiomer. Further conditions and abbreviations of CyDs can be found in Section 3.1 and Section 3.5.

Following CyD screening, we aimed to determine the enantiomer migration order (EMO), which was achieved using pure enantiomers isolated via HPLC (see Section 2.2). Unlike in HPLC, EMO reversal can occur in CE even when the stability constants of the (*S*)- and (*R*)-enantiomer complexes with CyDs are identical. This phenomenon arises because enantiomer discrimination in CE is influenced not only by differences in stability constants but also by variations in complex mobilities or a combination of both factors [50], as demonstrated by our results in Section 2.3 by CE. As shown in Table 2, multiple cases of EMO reversal were observed, highlighting an important result and the benefit of applying CyD-mediated CE. Although the migration order of NOR enantiomers remained identical regardless of the cavity size of the CyD used, cavity size-dependent EMO reversals were observed for LAU, Br-LAU, and PROP (see Table 2). The EMO is *R*,*S* for various β-CyD analogues but reversed for γ-CyDs, as shown by comparing HP-β-CyD with HP-γ-CyD, SBE-β-CyD with SBE-γ-CyD, and SBX with SGX. Figure 3 demonstrates examples of selector cavity size-dependent EMO reversals: when applying S-β-CyD, the first migrating peak for LAU is (*R*)-LAU, whereas with S-γ-CyD, the first peak corresponds to (*S*)-LAU, and a similar trend was observed for Br-LAU with SBX and SGX.

Substituent-dependent changes in EMO were observed when comparing CyDs of the same cavity size but with different substituents. For LAU, EMO reversal occurred when the CyD was carboxymethylated instead of sulfated. For sulfated or sulfoalkylated α- and β-CyDs (S-α-CyD, S-β-CyD, HDMS, and SBE-β-CyD), the migration order is *R*,*S*, but with carboxymethylated sidechains (CM-α-CyD and CM-β-CyD) the order reverses to (*S*) before (*R*)-LAU. This trend is also observed with NOR for both α-CyDs and γ-CyDs, when comparing S-α-CyD and HxDMS, with CM-α-CyD, as well as with S-γ-CyD and ODMS, but not with CM-γ-CyD (see Table 2).

**Figure 4:**
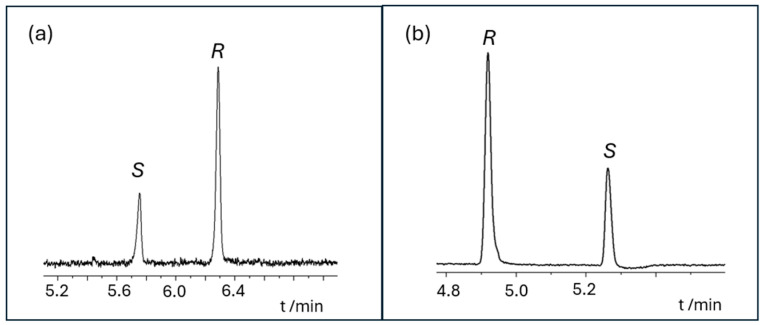
Demonstrative electropherograms depicting the substituent-dependent EMO reversal for LAU comparing (**a**) S-γ-CyD and (**b**) ODMS. Racemic samples were spiked with the (*R*)-LAU. Further conditions and abbreviations of CyDs can be found in Section 3.1 and Section 3.5.

When comparing results obtained with randomly sulfated and single-isomer CyDs, EMO changes were observed depending on the substitution pattern. For example, with Br-LAU and LAU, the (*S*)-enantiomer migrated first with randomly sulfated S-γ-CyD, but with all single-isomer selectors sulfated only on the primary side of the CyD rim, the (*R*)-enantiomer migrated first (see Figure 4). The additional modifications (methylation or acetylation) on the secondary side of the selector did not further alter the EMO. For single-isomer CyDs, the migration sequence remained nearly identical (*R*,*S*) across the analytes when comparing one CyD to another (such as SGX and single-isomer sulfated CyDs HxDMS, HDMS, ODMS, HS-β-CyD, and HDAS), except for SBX.

From the perspective of the analytes, although they are structurally very similar, they exhibited slight differences in EMO. For example, with S-α-CyD, all analytes followed the (*R*) before (*S*) order, except for PROP. In contrast, with S-γ-CyD, LAU and Br-LAU the order is (*S*) before (*R*), while NOR and PROP followed the reversed order. These observations suggest that more than one moiety (or specific part of the benzyltetrahydroisoquinoline skeleton) may be involved in chiral recognition. To further investigate and to clarify the underlying interactions, NMR experiments were conducted.

### 2.5. Characterization of the Inclusion Complexes by NMR Spectroscopy

To complement the CE experiments, NMR measurements were conducted to provide molecular-level insights into the interactions between the analytes and CyD selectors. Based on the CE results, the complex formation between SGX and LAU was further analyzed using NMR.

**Figure 5:**
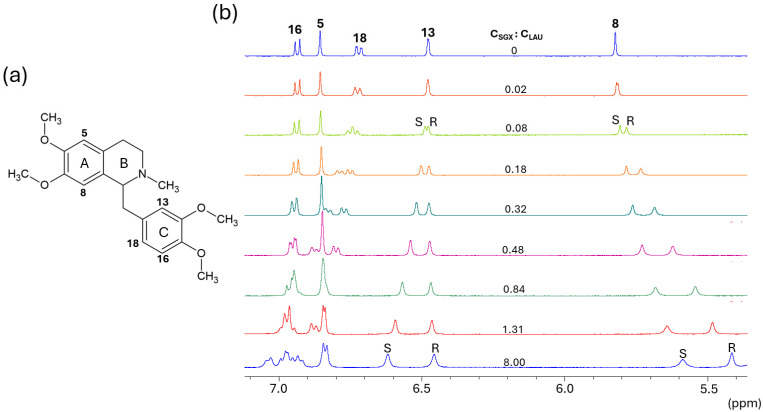
(**a**) Chemical structure and atom numbering of LAU, along with (**b**) selected ^1^H NMR spectra recorded at varying selector-to-analyte ratio in pD 7.4 phosphate buffer. Further conditions can be found in Section 3.6.

The ^1^H resonances of LAU were assigned in a pD 7.4 phosphate buffer solution (see Supplementary Table S3). Upon addition of SGX to the LAU sample diastereomeric splitting was observed. Figure 5 shows selected spectra from the ^1^H NMR titration of LAU with SGX. As evidenced by the recorded spectra, significant chemical shift changes occurred for the H8, H13, and H18 resonances of the LAU enantiomers, even at a 1:50 selector-to-analyte ratio. As the diastereomeric splitting increased with increasing selector concentration and exceeded 0.15 ppm for the H8 and H13 aromatic resonances above the 1:1 selector:analyte ratio, it may also serve as an analytical method to determine enantiomeric excess (see Figure 5 and in the Supplementary Materials Figure S9). Additional NMR signals from LAU, including methoxy protons (H20, H22, H24, and H26), as well as the *N*-methine (H10) and *N*-methyl (H27) resonances, also displayed significant splitting at higher selector concentrations (see Supplementary Figures S10 and S11). A similar phenomenon was observed for other LAU derivatives, NOR and PROP, supporting the possible involvement of both aromatic rings (A and C) in the interaction with the selector. Notably, for Br-LAU, where ring C has a significantly larger space due to the presence of a bromo substituent, the H13 proton exhibited the most pronounced chemical shift changes (see Supplementary Figure S12).

**Figure 6:**
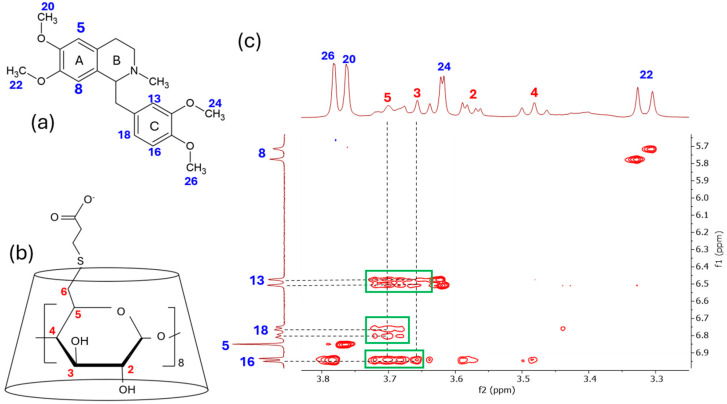
(**a**) Chemical structure and atom numbering of LAU, along with (**b**) the schematic representation and numbering of SGX. (**c**) Partial 2D ROESY NMR spectrum of the LAU-SGX (4:1) complex recorded in a 30 mM pD 7.4 phosphate buffered D_2_O, highlighting intermolecular interactions between SGX H3, H5 and LAU H13, H16, and H18 signals (green rectangle). Further conditions can be found in Section 3.6.

To investigate the spatial proximity between the protons of the SGX and LAU, 2D ROESY NMR experiments were performed. In the LAU:SGX 1:1 molar ratio sample, the H3 and H5 signals of SGX overlapped with the H24 methoxy signal of LAU. A similar overlap was observed in the 2D ROESY spectrum of NOR (see Supplementary Materials Figures S13 and S14). This overlap hindered the confident differentiation between intermolecular and intramolecular correlations in the ROESY spectrum. To address this issue, the experimental conditions were adjusted. In a sample with a SGX:LAU 0.25:1 ratio, distinct cross-peaks were observed for the H13, H16, and H18 resonances of LAU with the H5 resonance of SGX located within its cavity (see Figure 6). These findings clearly confirmed the immersion of ring C into the SGX cavity, furthermore, the cross-peak between SGX H3 proton and LAU H13, H16 suggests that C ring of LAU immersed from the secondary side of SGX. Additionally, cross-peaks were observed between the H5 proton of LAU exhibited spatial proximity to the CH_2_ side-chain signals of SGX suggesting that ring A is located near the primary side of SGX rather than inside the SGX cavity. This interpretation is further supported by the cross-peaks between the H7 and H8 protons of SGX and the H20 methoxy proton of LAU (see Supplementary Material, Figure S15). Furthermore, the absence of ROESY cross-peaks between the protons of LAU’s ring A and the SGX inner cavity protons confirms that ring A does not reside within the SGX cavity.

For the Br-LAU–SGX and PROP–SGX complexes, the H5 proton of SGX was resolved from the H24 methoxy signal, allowing the clear identification of the spatial proximity between the SGX H5 proton and the aromatic protons of ring C of these analytes (see Supplementary Materials Figures S16 and S17). These findings suggest that the structural modifications of LAU, such as the presence of a bromo substituent on the C ring, *N*-demethylation, or an *N*-propyl group on the B ring do not alter the location of the C ring within the SGX cavity.

Based on the CE results, a cavity size-dependent EMO reversal was observed for Br-LAU when comparing SBX and SGX. To investigate this phenomenon at the atomic level, the Br-LAU–SBX and Br-LAU–SGX complexes were further analyzed using ^1^H and 2D ROESY NMR spectroscopy. A comparison of the ^1^H NMR spectra for the Br-LAU–SBX and Br-LAU–SGX complexes revealed that the H13 resonance exhibited more pronounced diastereomeric splitting with SGX. In the 2D ROESY NMR spectra, contrary to the confirmed inclusion complex formation between Br-LAU and SGX, no spatial proximity was detected between the cavity protons (H3 and H5) of SBX and Br-LAU, suggesting an outer-sphere interaction rather than an inclusion-type complex. The presence of the bromine substituent on the C ring may result in the beta-cavity not being of sufficient size for inclusion. This observation is further supported by the lower complex stability values measured by CE for the Br-LAU–SBX complex compared to the Br-LAU–SGX complex (see Supplementary Materials Figures S17–S19 and Table S2). These results demonstrate that the mechanism of complex formation depends on the spatial properties of the immersed part of the ligand and differs between these two, different cavity sized selectors.

## 3. Materials and Methods

### 3.1. Materials

Native CyDs (α, β and γ-CyD) and their derivatives, RAME-γ-CyD with the average degree of substitution (DS)~12, dimethylated-β-CyD DS~14 (DIME-β-CyD), hydroxypropylated-α-CyD DS~3 (HP-α-CyD), HP-β-CyD DS~3 and hydroxypropylated-γ-CyD DS~3 (HP-γ-CyD), carboxymethylated-α-CyD DS~3.5 (CM-α-CyD), CM-β-CyD DS~3, CM-γ-CyD DS~4, sualfadex (SAX, hexakis-(6-deoxy-6-(2-carboxyethyl)-thio)-α-CyD), subetadex (SBX, heptakis-(6-deoxy-6-(2-carboxyethyl)-thio)-β-CyD), sugammadex (SGX, octakis-(6-deoxy-6-(2-carboxyethyl)-thio)-γ-CyD), sulfobutylether-α-CyD DS~4 (SBE-α-CyD), sulfobutyl-ether-β-CyD, DS~6.5 (SBE-β-CyD), sulfobutyl-ether-γ CyD DS~4 (SBE-γ-CyD), sulfopropylated-β-CyD DS~4 (SP-β-CyD), sulfopropylated-γ-CyD DS~2 (SP-γ-CyD), sulfated-α-CD DS~12 (S-α-CyD), S-β-CyD DS~13, S-γ-CyD DS~14, heptakis-(6-*O*-sulfo)-β-CyD (HS-β-CyD), heptakis-(2,3-*O*-dimethyl-6-*O*-sulfo)-β-CD (HDMS), heptakis-(2,3-*O*-diacethyl-6-*O*-sulfo)-β-CyD (HDAS), heptakis-(2-*O*-methyl-3,6-*O*-disulfo)-β-CyD (HMDiSu), hexakis-(2,3-*O*-dimethyl-6-*O*-sulfo)-α-CyD (HxDMS), octakis-(2,3-*O*-dimethyl-6-*O*-sulfo)-γ-CyD (ODMS) were products of CarboHyde Ltd. (Budapest, Hungary) and CycloLab Ltd. (Budapest, Hungary). D_2_O (99.9% D) was purchased from Merck (Darmstadt, Germany).

Polysaccharide-type chiral columns: Chiralpak AD, containing amylose tris(3,5-dimethylphenylcarbamate), Chiralcel OD and Chiralpak IA containing cellulose tris(3,5-dimethylphenylcarbamate, however, the former contains the coated derivatized polysaccharide while in the later the polymer is immobilized on the silica matrix. Chiracel OD, Chiralpak AD, Chiralpak IA were products of Daicel (Tokyo, Japan). CyD-based chiral columns: Chiral CD-pH, containing phenylcarbamate-β-cyclodextrin; Cyclobond I containing native β-CyD; and Nucleodex β-PM containing permethylated β-CyD as the chiral selector. Astec Cyclobond I 2000 and Shisedio Chiral CD-Ph were products of Sigma-Aldrich, (St. Louis, MO, USA), while Nucleodex Beta-PM and Shisedio Chiral CD-Ph were purchased from Phenomenex (Torrance, CA, USA).

Gradient grade MeOH was purchased from Merck (Darmstadt, Germany) and diethylamine (DEA) was ordered from Sigma-Aldrich (Budapest, Hungary).

Sodium dihydrogen phosphate (NaH_2_PO_4_), sodium hydroxide (NaOH), MeOH, and dimethyl sulfoxide (DMSO) used for the preparation of buffer solutions, rinsing solutions, or applied as sample solvent or EOF marker were of analytical grade and purchased from commercial suppliers (Sigma-Aldrich, Budapest, Hungary). Bidistilled Millipore water was used throughout this study.

(*S*)-NOR was provided by Prof. Jean-Pierre Hurvois. 3,4-dihydropapaverine was a gift from Chinoin-Sanofi Co., Budapest. Melting points were determined in open capillary tubes and are uncorrected. Thin layer chromatography (TLC): Al sheets Kieselgel 60 F254 (Merck, Darmstadt, Germany) thickness of layer 0.2 mm.

### 3.2. Syntheses of Benzyltetrahydroisoquinoline Alkaloids

#### 3.2.1. Synthesis of Racemic NOR

To a stirred solution of 3,4-dihydropapaverine (6 g, 0.018 mol) in MeOH (100 mL), sodium borohydride (3 g, 0.079 mol) was added in small portions at 5 °C. After stirring for 2 h at room temperature, the solvent was evaporated under vacuum. The residue was diluted with water and extracted with chloroform. The organic layer was washed with brine, dried over sodium sulfate, and evaporated, yielding NOR as an oil. This oil was dissolved in ethanol and acidified with ethanol saturated with HCl gas. The resulting hydrochloride salt (4.6 g, 0.012 mol, 67% yield) was obtained with a melting point of 218–219 °C. The complete ^1^H and ^13^C NMR resonance assignments of NOR in CDCl_3_ along with the atomic numbering can be found in the Supplementary Materials Figures S20–S24, and Tables S4 and S5.

#### 3.2.2. Synthesis of Racemic LAU

NOR base (2.5 g, 0.007 mol) was dissolved in MeOH (40 mL), and 37% formaldehyde (8 mL, 9.8 g, 0.121 mol,) was added. The reaction mixture was stirred for 1 h, followed by the addition of sodium borohydride under ice bath conditions. After stirring at room temperature for 8 h (monitored by TLC: chloroform-MeOH 9:1 (*v*/*v*), chloroform–acetone–diethyl amine 5:4:1 (*v*/*v*)), the reaction was quenched with saturated ammonium chloride solution. MeOH was evaporated under reduced pressure, and the residue was suspended in water. The solid was filtered, dried, and crystallized from ethanol–hexane, yielding 1.6 g (0.004 mol, 57% yield) of LAU with a melting point of 115–117 °C. The complete ^1^H and ^13^C NMR resonance assignments of LAU in CDCl_3_ can be found in the Supplementary Materials Figures S25–S28, and Tables S4 and S5.

#### 3.2.3. Synthesis of Racemic PROP

A mixture of NOR (1.7 g, ~5 mmol), sodium bicarbonate (1.2 g, 0.014 mol), and propyl bromide (0.55 mL, 0.41 g, 0.003 mol) were dissolved in dimethylformamide (30 mL, 31.78 g, 0.435 mol) and were stirred at 80 °C for 16 h. After filtering the inorganic salts, the filtrate was evaporated under reduced pressure. The residue was partitioned between chloroform and 5% ammonia solution. The aqueous phase was extracted with chloroform, and the combined organic phases were washed with brine, dried over sodium sulfate, and evaporated. The residue was subjected to silica gel column chromatography, using chloroform-MeOH (9:1, *v*/*v*) as the eluent. 1.6 g (0.004 mol, 80% yield) oily PROP was obtained. Subsequently it was dissolved in ethanol, and 30% HBr in acetic acid was added. The HBr salt of PROP crystallized with a melting point of 214–216 °C. The complete ^1^H and ^13^C NMR resonance assignments of LAU in CDCl_3_ can be found in the Supplementary Materials Figures S29–S32, and Tables S4 and S5.

#### 3.2.4. Synthesis of Racemic Br-LAU

A solution of bromine (2.0 g, 0.65 mL, 0.025 mol) in acetic acid (30 mL) was added dropwise to an ice-cooled solution of racemic LAU (3.57 g, 0.010 mol) and sodium acetate (1.35 g, 0.016 mol) in 25% aqueous acetic acid (150 mL). The mixture was stirred for an additional 2 h, during which the yellow precipitate dissolved. After the addition of 10% NaOH, the product was extracted with ether. The organic phase was dried and evaporated. The residue was crystallized from ethanol, yielding 2.8 g (0.006 mol, 60% yield) of product with a melting point of 124–125 °C, matching the literature value [51]. The ^1^H and ^13^C NMR resonances of racemic Br-LAU were assigned based on conventional 1D and 2D NMR experiments (see in the Supplementary Materials Figures S33–S36, and Tables S4 and S5).

### 3.3. Chiral HPLC

The chiral HPLC experiments were carried out on Agilent 1100 HPLC system, consisting of an inline degasser (G1322A), a quaternary pump (G1311A), an automatic injector (G1329A) paired with sample thermostat (G1330A), a column thermostat (G1316A), and a diode array detector (G1315A) with Agilent Chemstation B04.03-SP2 software (Agilent, Bronnwald, Germany). Chromatographic analysis was performed at 25 °C with a mobile phase flow rate of 0.7 mL/min in polar organic mode.

The chromatography screening study was performed at 25 °C on polysaccharide type Chiracel OD, Chiralpak AD, Chiralpak IA with identical dimensions (250 × 4.6 mm; particle size 10 µm) and CD-based, Astec Cyclobond I 2000 (250 × 4.6 mm; particle size 10 µm), Nucleodex Beta-PM (200 × 4.0 mm; particle size 5 µm), Chiral CD-Ph (250 × 4.6 mm; particle size 10 µm) chiral columns with a mobile phase flow rate 0.7 mL/min. Isocratic elution was applied using ACN:DEA 100:0.1 and MeOH:DEA 100:0.1 as eluent. The injection volume was 2 µL. The detection wavelength was 270 nm. Sample preparation: 1 mg/mL stock solution in MeOH.

For semi-preparative scale separation of PROP Chiralpak AD column (10 µm, 250 × 10 mm), while for semi-preparative separation of LAU and Br-LAU Chiralcel OD column (10 µm, 250 × 10 mm) was used using uniformly MeOH:DEA 100:0.1 eluent at 30 °C.

The injection volume was 20 µL using 100 parallel measurements. Sample preparation: 20 mg/mL stock solution in MeOH.

### 3.4. CD Spectroscopy

Circular Dichroism experiments were conducted on a Jasco J-810 spectropolarimeter (Japan Spectroscopic Co., Tokyo, Japan). The concentration of the enantiomeric stock solutions was 0.3 mg/mL. The samples measured in quartz cells of 1 mm path length. CD spectra were collected at 25 °C. In total, 5 scans were accumulated in the 220–325 nm wavelength range. The CD spectrometer was set to continuous scanning mode 50 nm/min scanning speed, 1 nm bandwidth, 0.2 nm data pitch, and the applied response time was 4 s. Baseline correction and Savitzky–Golay smoothing were applied.

### 3.5. Capillary Electrophoresis

The CE experiments were conducted using an Agilent 7100 instrument (Agilent Technologies, Waldbronn, Germany) equipped with a photodiode array detector and the Chemstation software for data processing. Untreated fused silica capillaries (50 μm id, 48.5 cm total, 40 cm effective length) were purchased from Agilent. The conditioning of new capillaries involved sequential flushing with 1 M NaOH, followed by 0.1 M NaOH, and finally water, each for 30 min. Before each run, the capillary was preconditioned by rinsing with the BGE for 2 min. The temperature of the capillary was set to 25 °C. UV detection was performed at a wavelength of 200 nm, and 15 kV voltage was used during the measurements. Samples were injected hydrodynamically at 150 mbar·sec. The running buffers consisted of 30 mM phosphate buffer adjusted to pH 7.4 with 1 M NaOH. The BGE contained CyDs at various concentrations (0.25–30 mM). For the screening experiments, each racemic alkaloid was prepared separately in MeOH (1 mg/mL), and appropriate dilutions with water were used to prepare sample solutions. For the determination of EMO, spiked samples were used. In the apparent complex stability constant determination studies DMSO (0.1%) was applied as an EOF marker. CEVal software (v. 0.6i3) was used to evaluate the apparent complex stability constants [52]. To characterize the affinity of the interaction in CE, the initial step is to calculate the effective mobility of the analyte at different CyD concentrations. Affinity CE measurements often show triangular peaks as a result of electromigration dispersion. These biases can be alleviated by using the HVL function (1), which gives a more accurate estimate of the effective mobility.

(1) $${HVL}_{\delta }\left(t;a_{0},a_{1},a_{2},a_{3\delta }\right)=\frac{\frac{a_{0}}{a_{2}a_{3\delta }\sqrt{2\pi }}exp\left[{-\frac{1}{2}\left(\frac{t-a_{1}}{a_{2}}\right)}^{2}\right]}{\frac{1}{exp\left(a_{3\delta }\right)-1}+\frac{1}{2}\left[1+erf\left(\frac{t-a_{1}}{\sqrt{2a_{2}}}\right)\right]}$$

where *a*_0_ is the area of the HVL function, *a*_1_ is the position of the Gaussian component corresponding to the migration time of the analyte, *a*_2_ is the standard deviation of the Gaussian component, and *a*_3*δ*_ is the triangular distortion.

Assuming a 1:1 complexation ratio and that the equilibrium CyD concentration is almost equal to the total CyD concentration, the complex stability constant can be described by the function (2).

(2) $$\mu_{eff}=\frac{\mu_{A}+\mu_{ACyD}K_{stab}\left[CyD\right]}{1+K_{stab}\left[CyD\right]}$$

where *μ*_*eff*_ is the effective mobilities of the analyte, *μ*_*A*_ and *μ*_*A* CyD_ are the free and complexed analyte mobilities, [*C*y*D*] is the concentration of the selector, and *K*_*stab*_ is the complex stability constant. To calculate the exact apparent, averaged complex stability constant values, viscosity correction [53], and ionic strength correction [54] were applied.

The *pKa* prediction was performed using the ChemAxon online platform (Budapest, Hungary).

### 3.6. NMR Spectroscopy

The NMR experiments were carried out at 298 K on a Bruker Avance III (^1^H: 500 MHz, ^13^C: 125 MHz) Spectrometer and a Bruker Avance III (^1^H: 400 MHz, ^13^C: 100 MHz) Spectrometer. Each pulse program was taken from the available TopSpin software library (v. 3.6.2). Conventional 2D experiments (^1^H-^1^H COSY, ^1^H-^1^H ROESY, ^1^H-^13^C HSQC, ^1^H-^13^C HMBC) for structural elucidation were acquired on 30 mM phosphate buffer pH 7.4 solutions containing 10 mM of the alkaloids and 10 mM SGX (resulting in a 1:1 analyte–CyD molar ratio). Spiked samples were prepared as follows, 1:3 molar ratio of (*R*)-enantiomer and (*S*)-enantiomer respectively. ROESY experiments were recorded with a 300 ms mixing time.

## 4. Conclusions

This study provides a comprehensive analysis of the enantiomeric separation of benzyltetrahydroisoquinoline alkaloids utilizing CE and HPLC. Through extensive screening with 21 CyDs, as chiral selectors in CE, we achieved multiple baseline enantioseparations, demonstrating that anionic CyDs are especially well-suited for the chiral analysis of these alkaloids. Among the investigated chiral HPLC stationary phases, polysaccharide-based columns demonstrated superior performance compared to CyD-based ones. Notably, Chiralpak AD proved to be more effective than Chiralpak IA, even though both utilize the same selector. This improved efficiency likely stems from variations in the silica immobilization process. Following analytical-scale HPLC separations, enantiomers were successfully isolated on a semi-preparative scale, enabling the determination of EEO in HPLC and EMO in CE. Particular attention was given to the observation of EMO reversal, which was found to depend on the cavity size, substituent nature, and substitution pattern of the CyDs. In general, the (*R*)-enantiomer tended to migrate first with anionic CyDs, whereas the carboxymethylated and sugammadex derivatives more commonly exhibited an *S*,*R* migration order.

The CyD-based chiral separations were complemented by NMR spectroscopic studies. In the presence of SGX, diastereomeric recognition was confirmed by the splitting of all LAU signals in the ^1^H NMR spectra. 2D ROESY results confirm the inclusion complex formation of LAU derivatives, where the structural modifications of LAU do not alter the type of immersion into the SGX cavity. The bromination of the ring C resulted in a remarkable change in the encapsulated part of the ligand, which requires a gamma cavity size for inclusion. These findings enhance the understanding of enantiomeric separation mechanisms and provide practical insights into the selection of CyDs for chiral separation of benzyltetrahydroisoquinoline alkaloids. This study furthermore emphasizes the importance of the close cooperation of CE, HPLC, and NMR in advancing enantiomer separation methods, offering a robust framework for both analytical and preparative applications in pharmaceutical field.

## Figures and Tables

**Table 1:** Retention times of the first enantiomer (t_1_), resolution values (R_s_), and enantiomer elution order (EEO) of LAU derivatives applying six different analytical chiral column, methanol (MeOH), or acetonitrile (ACN) as mobile phase with 0.1% diethylamine (DEA). Further conditions can be found in Section 3.3. Chiral HPLC.

Column	Mobile Phase	LAU			NOR			Br-LAU			PROP		
		t_1_	*R_s_*	EEO	t_1_	*R_s_*	EEO	t_1_	*R_s_*	EEO	t_1_	*R_s_*	EEO
Chiral CD-Ph	ACN	5.39	-	-	5.76	-	-	9.21	-	-	5.79	-	-
	MeOH	11.85	-	-	12.17	0.8	*S*,*R*	9.53			8.16	-	-
Cyclobond I	ACN	7.79	-	-	8.48	-	-	6.34	-	-	5.04	-	-
	MeOH	5.11	-	-	5.10	-	-	4.97	-	-	4.79	-	-
Nucleodex β-CD	ACN	2.68	-	-	2.69	-	-	2.70	-	-	2.68	-	-
	MeOH	2.87	-	-	3.21	-	-	2.88	-	-	2.88	-	-
Chiralcel OD	ACN	5.44	2.7	*S*,*R*	7.33	2.9	*S*,*R*	5.68	2.4	*S*,*R*	5.17	0.8	*S*,*R*
	MeOH	7.00	3.2	*S*,*R*	7.74	1.0	*S*,*R*	7.17	2.8	*S*,*R*	6.89	1.8	*S*,*R*
Chiralpak AD	ACN	4.87	-	-	5.91	3.7	*S*,*R*	5.00	-	-	4.80	-	-
	MeOH	5.92	0.6	*S*,*R*	6.98	4.7	*S*,*R*	5.87	1.9	*S*,*R*	6.50	1.9	*R*,*S*
Chiralpak IA	ACN	5.10	0.5	*S*,*R*	6.37	3.6	*S*,*R*	5.28	-	-	5.03	-	-
	MeOH	6.37	-	-	6.72	3.0	*S*,*R*	6.45	-	-	6.39	0.9	*R*,*S*

**Table 2:** The maximum resolution values (*R_s_*) and enantiomer migration order (EMO) of LAU derivatives applying various CyDs at 30 mM phosphate buffer (pH 7.4), 25 °C, 15 kV, 200 nm. Further conditions and abbreviations of CyDs can be found in Section 3.1 and Section 3.5.

		CyD		LAU	NOR	Br-LAU	PROP
Neutral CyDs	Randomly substituted	HP-β-CyD	EMO	-	-	*R*,*S*	-
			*R_s_*	-	-	0.8 (30 mM)	-
		HP-γ-CyD	EMO	*S*,*R*	-	*S*,*R*	*S*,*R*
			*R_s_*	0.4 (30 mM)	-	0.4 (30 mM)	1.3 (30 mM)
Anionic CyDs		S-α-CyD	EMO	*R*,*S*	*R*,*S*	*R*,*S*	*S*,*R*
			*R_s_*	2.6 (10 mM)	2.1 (10 mM)	0.9 (10 mM)	0.4 (10 mM)
		S-β-CyD	EMO	*R*,*S*	*R*,*S*	*R*,*S*	*R*,*S*
			*R_s_*	3.4 (10 mM)	7.5 (10 mM)	0.7 (10 mM)	5.4 (10 mM)
		S-γ-CyD	EMO	*S*,*R*	*R*,*S*	*S*,*R*	-
			*R_s_*	10.5 (10 mM)	5.4 (10 mM)	7.3 (10 mM)	-
		SP-α-CyD	EMO	-	-	-	*S*,*R*
			*R_s_*	-	-	-	0.7 (10 mM)
		SP-β-CyD	EMO	-	-	-	*S*,*R*
			*R_s_*	-	-	-	0.7 (10 mM)
		SP-γ-CyD	EMO	*S*,*R*	-	*S*,*R*	*S*,*R*
			*R_s_*	1.2 (10 mM)	-	1.5 (10 mM)	2.1 (10 mM)
		SBE-α-CyD	EMO	-	-	*S*,*R*	-
			*R_s_*	-	-	0.5 (10 mM)	-
		SBE-β-CyD	EMO	*R*,*S*		*R*,*S*	
			*R_s_*	1.0 (10 mM)	-	2.3 (10 mM)	-
		SBE-γ-CyD	EMO	-	-	*S*,*R*	*S*,*R*
			*R_s_*	-	-	1.1 (10 mM)	1.3 (10 mM)
		CM-α-CyD	EMO	*S*,*R*	*S*,*R*	-	*S*,*R*
			*R_s_*	0.6 (10 mM)	1.0 (10 mM)	-	4.8 (10 mM)
		CM-β-CyD	EMO	*S*,*R*	-	-	*S*,*R*
			*R_s_*	0.7 (10 mM)	-	-	1.0 (10 mM)
		CM-γ-CyD	EMO	*S*,*R*	*S*,*R*	*S*,*R*	*S*,*R*
			*R_s_*	3.2 (7.5 mM)	1.0 (10 mM)	3.3 (7.5 mM)	2.7 (10 mM)
		SBX	EMO	*S*,*R*	*S*,*R*	*R*,*S*	-
	Single isomer		*R_s_*	2.6 (7.5 mM)	3.3 (7.5 mM)	2.9 (7.5 mM)	-
		SGX	EMO	*S*,*R*	*S*,*R*	*S*,*R*	*S*,*R*
			*R_s_*	6.7 (5 mM)	5.7 (5 mM)	2.2 (2.5 mM)	7.2 (2.5 mM)
		HS-β-CyD	EMO	*R*,*S*	*R*,*S*	*R*,*S*	*R*,*S*
			*R_s_*	1.5 (4 mM)	0.6 (5 mM)	1.8 (4 mM)	2.1 (5 mM)
		HDAS	EMO	*R*,*S*	*R*,*S*	*R*,*S*	*R*,*S*
			*R_s_*	8.3 (2 mM)	3.2 (2 mM)	5.0 (2 mM)	4.7 (2 mM)
		HxDMS	EMO	*R*,*S*	*R*,*S*	*R*,*S*	*R*,*S*
			*R_s_*	2.2 (5 mM)	1.0 (5 mM)	1.9 (5 mM)	0.5 (5 mM)
		HDMS	EMO	*R*,*S*	*R*,*S*	*R*,*S*	*R*,*S*
			*R_s_*	7.0 (3 mM)	6.1 (2 mM)	5.6 (3 mM)	8.3 (3 mM)
		ODMS	EMO	*R*,*S*	*R*,*S*	*R*,*S*	*R*,*S*
			*R_s_*	2.8 (4 mM)	7.5 (2 mM)	6.3 (2 mM)	1.8 (2 mM)

## Data Availability

Data are contained within the article and Supplementary Materials.

## Supplementary Materials

The following supporting information can be downloaded at: https://www.mdpi.com/article/10.3390/molecules30051125/s1, Figure S1. (a) HPLC chromatogram showing the separation of the enantiomers of Br-LAU using a Chiralpak AD column with methanol:diethylamine (MeOH:DEA) 100:0.1 as the eluent system. (b) HPLC chromatogram using a Chiralcel OD column under the same eluent conditions. Further conditions can be found in Section 3.3. Chiral HPLC. Figure S2. (a) HPLC chromatogram showing the partial separation of the enantiomers of LAU using a Chiralpak AD column with methanol:diethylamine (MeOH:DEA) 100:0.1 as the eluent system. (b) HPLC chromatogram using a Chiralcel OD column under the same eluent conditions. Further conditions can be found in Section 3.3. Chiral HPLC. Figure S3. (a) HPLC chromatogram showing the separation of the enantiomers of NOR using a Chiralpak AD column with methanol:diethylamine (MeOH:DEA) 100:0.1 as the eluent system. (b) HPLC chromatogram using a Chiralcel OD column under the same eluent conditions. Further conditions can be found in Section 3.3. Chiral HPLC. Figure S4. HPLC chromatograms showing the separation of the enantiomers of PROP using a (a) Chiralpak AD column and a (b) Chiralcel OD column with methanol:diethylamine (MeOH:DEA) 100:0.1 as the eluent system. Further conditions can be found in Section 3.3. Chiral HPLC. Figure S5. CD spectra of (S)-NOR. (0.3 mg/mL in MeOH). Further conditions can be found in Section 3.4. CD spectroscopy. Figure S6. CD spectra of (S)-LAU (grey) and (R)-LAU (red). (0.3 and 0.5 mg/mL, respectively, in MeOH). Further conditions can be found in Section 3.4. CD spectroscopy. Figure S7. CD spectra of (S)-Br-LAU (grey) and (R)-Br-LAU (red). (0.3 mg/mL in MeOH). Further conditions can be found in Section 3.4. CD spectroscopy. Figure S8. CD spectra of (S)-PROP (grey) and (R)-PROP (red). (0.3 mg/mL in MeOH). Further conditions can be found in Section 3.4. CD spectroscopy. Table S1. Some averaged apparent LAU-CyD complex stability constants (K_stab_/M^−1^) and complex mobilities (µ_(AS)_/10^−^^5^ cm^2^V^−^^1^s^−^^1^) of the transient diastereomeric complexes measured by affinity capillary electrophoresis at 30 mM phosphate buffer (pH 7.4), 25 °C, 15 kV, 200 nm. Further conditions and CyD abbreviations can be found in Section 3.5. Capillary Electrophoresis, 3.1. Materials. Table S2. Averaged apparent alkaloid–CyD complex stability constants (K_stab_/M^−1^) and complex mobilities (µ_(AS)_/10^−^^5^ cm^2^V^−^^1^s^−^^1^) measured by affinity capillary electrophoresis at 30 mM phosphate buffer (pH 7.4), 25 °C, 15 kV, 200 nm. Further conditions and CyD abbreviations can be found in Section 3.5. Capillary Electrophoresis, 3.1. Materials. Table S3. Complete ^1^H NMR resonances assignment for LAU in D_2_O (30 mM phosphate buffer pD 7.4; 298 K; 500 MHz). Figure S9. (a) H8 and H13 aromatic protons of LAU. (b) LAU in 30 mM phosphate buffer in D_2_O pD 7.4. (c) SGX:LAU = 0.38:1 complex and (d) spiked LAU-SGX = 1:1 complex in the same buffer system. The samples were spiked with (S)-enantiomers, 30 mM phosphate buffer in D_2_O pD 7.4 solutions containing 10 mM of the alkaloids and 10 mM SGX. Figure S10. (a) The structure of LAU, labelled its chiral center (H10 proton). (b) Part of ^1^H NMR spectra of LAU in pD 7.4 phosphate buffer ^1^H titration recorded at 500 MHz, denoted the H10 protons of (S)- and (R)-LAU. Figure S11. (a)The structure of LAU, labelled its aliphatic protons. (b) The structure of SGX, numbered its H2-H8 protons. Part of ^1^H NMR spectra of LAU in pD 7.4 phosphate buffer ^1^H titration recorded at 500 MHz, denoted the protons of LAU (blue), SGX (red). Figure S12. (a) H8 and H13 aromatic protons of LAU derivatives. (R^1^ = R^2^= H NOR, R^1^= CH_3_ R^2^= H LAU, R^1^ = CH_3_ R^2^= Br Br-LAU, R^1^ = CH_2_CH_2_CH_3_ R^2^= H PROP), Part of the ^1^H NMR spectra (aromatic region) of alkaloid-SGX complexes; (b) PROP-SGX = 1:1, (c) NOR-SGX = 2:1, (d) Br-LAU-SGX = 1:2, (e) LAU-SGX = 1:1. Figure S13. (a) The structure of LAU, labelled H5, H8, H13, H20, H22, H24 and H26 protons of them. (b) Schematic representation of SGX and its H2, H3, H4, H5 and H6 protons. (c) Partial 2D ROESY NMR spectrum of the LAU-SGX (1:1) complex. The intramolecular interaction between H16, H18 and H5 protons of SGX (in the green box). The intermolecular interaction between aromatic and methoxy signals of LAU (in the purple box). Intermolecular and intramolecular interactions might overlap (in the red box). The sample was spiked with (S)-LAU, 30 mM phosphate buffer in D2O pD 7.4 solution, recorded at 400 MHz. Figure S14. (a) The structure of NOR, labelled H5, H8, H13, H20, H22, H24 and H26 protons of them. (b) H2, H3, H4 and H5 protons of SGX. (c) Partial 2D ROESY NMR spectrum of the NOR-SGX (2:1) complex, recorded at 400 MHz. The intermolecular interactions between aromatic and methoxy signals of NOR (in the purple box). The intramolecular interactions between SGX H3, H5 and H16, H18 signals of NOR (in the green box). Intermolecular and intramolecular interactions might overlap (in the red box). The sample was spiked with (S)-NOR, 30 mM phosphate buffer in D_2_O pD 7.4 solution. Figure S15. (a) Chemical structure and atom numbering of LAU, along with (b) the schematic representation and numbering of SGX. (c) Partial 2D ROESY NMR spectrum of the LAU-SGX (1:1) complex. The intramolecular interaction between H7, H8 protons of SGX and H5 proton of LAU. (d) Partial 2D ROESY NMR spectrum of the LAU-SGX (1:1) complex. The intramolecular interaction between H7, H8 protons of SGX and H20 proton of (S)- and (R)-LAU. Further conditions can be found in Section 3.6. NMR spectroscopy. Figure S16. (a) The structure of PROP, labelled H5, H8, H13, H16, H18 and H22 protons of PROP. (b) H2, H3, H4 and H5 protons of SGX. (c) Partial 2D ROESY NMR spectrum of the PROP-SGX complex, recorded at 500 MHz. The intramolecular interactions between H8 aromatic and H22 methoxy signals of PROP (in the purple box). The intermolecular interactions between SGX H3, H5 and H5, H8, H13, H16, H18 aromatic signals of PROP (in the green box and marked with dotted lines). Intermolecular and intramolecular interactions might overlap (in the red box). The sample was spiked with (S)-PROP, 30 mM phosphate buffer in D_2_O pD 7.4 solution. Figure S17. (a) The structure of Br-LAU and its H5, H8, H13 and H16 aromatic protons labelled with blue. (b) Schematic representation of SGX and its H2, H3, H4, H5, and H6 protons. (c) Partial 2D ROESY NMR spectrum of the Br-LAU-SGX (1:2.5) complex, recorded at 400 MHz, denoted the spatial proximity between SGX the H5 and the H13, H16 aromatic signals of Br-LAU (in the green box). Intramolecular interactions between H2a signal and H5 aromatic protons of Br-LAU (in the purple box). Intermolecular and intramolecular interactions may overlap (in the red box). The sample was spiked with (S)-Br-LAU, 30 mM phosphate buffer in D_2_O pD 7.4 solution. Figure S18. Partial 2D ROESY NMR spectrum of Br-LAU-SBX (1:2) complex, 30 mM phosphate buffer in D_2_O pD 7.4 solution. Figure S19. (a) The structure of Br-LAU and its H5, H8, H13 and H16 aromatic protons labelled with brown. ^1^H spectra of Br-LAU SBX complex and Br-LAU SGX complex. The sample was spiked with (S)-Br-LAU, 30 mM phosphate buffer in D_2_O pD 7.4 solution. Figure S20. The basic structure of LAU derivatives. R_1_ = CH_3_ and R_2_ = H Laudanosine (LAU), R_1_ = R_2_ = H Norlaudanosine (NOR), R_1_ = CH_2_CH_2_CH_3_ and R_2_ = H N-propyl-norlaudanosine (PROP), R_1_ = CH_3_ and R_2_ = Br 6′-Br-laudanosine (Br-LAU). Table S4. ^1^H and ^13^C assignment of LAU derivatives, (in CDCl_3_-d, 400 MHz). For the atomic positions of LAU derivatives see Figure S9. Table S5. ^1^H and ^13^C chemical shifts of propyl group of PROP (in CDCl_3_-d, 400 MHz). Figure S21. ^1^H NMR spectrum of racemic NOR. (in CDCl_3_, 298 K, 400 MHz). Figure S22. ^13^C NMR spectrum of racemic NOR. (in CDCl_3_, 298 K, 400 MHz). Figure S23. HSQC spectrum of racemic NOR. (in CDCl_3_, 298 K, 400 MHz). Figure S24. HMBC spectrum of racemic NOR. (in CDCl_3_, 298 K, 400 MHz). Figure S25. ^1^H NMR spectrum of racemic LAU. (in CDCl_3_, 298 K, 400 MHz). Figure S26. ^13^C DEPT NMR spectrum of racemic LAU. (in CDCl_3_, 298 K, 400 MHz). Figure S27. HSQC spectrum of racemic LAU. (in CDCl_3_, 298 K, 400 MHz). Figure S28. HMBC spectrum of racemic LAU. (in CDCl_3_, 298 K, 400 MHz). Figure S29. ^1^H spectrum of racemic PROP. (in CDCl_3_, 298 K, 400 MHz). Figure S30. ^13^C spectrum of racemic PROP. (in CDCl_3_, 298 K, 400 MHz). Figure S31. HSQC spectrum of racemic PROP. (in CDCl_3_, 298 K, 400 MHz). Figure S32. HMBC spectrum of racemic PROP. (in CDCl_3_, 298 K, 400 MHz). Figure S33. ^1^H NMR spectrum of racemic Br-LAU. (in CDCl_3_, 298 K, 400 MHz). Figure S34. ^13^C NMR spectrum of racemic Br-LAU. (in CDCl_3_, 298 K, 400 MHz). Figure S35. HSQC spectrum of racemic Br-LAU. (in CDCl_3_, 298 K, 400 MHz). Figure S36. HMBC spectrum of racemic Br-LAU. (in CDCl_3_, 298 K, 400 MHz).
