# Supplementary material for: Chiral Recognition Mechanism of Benzyltetrahydroisoquinoline Alkaloids: Cyclodextrin-Mediated Capillary Electrophoresis, Chiral HPLC, and NMR Spectroscopy Study
Source: Molecules. 2025 Feb 28;30(5):1125. doi: 10.3390/molecules30051125 (PMC11901523; doi:10.3390/molecules30051125)
Supplement: Supplementary file 1 [file molecules-30-01125-s001.zip › molecules-3474751-supplementary.pdf]

Supplementary Material

Chiral Recognition Mechanism of Benzyltetrahydroisoquinoline Alkaloids: Cyclodextrin-Mediated Capillary Electrophoresis, Chiral HPLC, and NMR Spectroscopy Study

Erzsébet Várnagy [1,2], Gergő Tóth [2,3], Sándor Hosztafi [2,3], Máté Dobó [2,3], Ida Fejős [1,2*], Szabolcs Béni [4*]

1 Department of Pharmacognosy, Semmelweis University, Üllői út 26, H-1085 Budapest, Hungary;
2 Center for Pharmacology and Drug Research & Development, Semmelweis University, Üllői út 26, H-1085 Budapest, Hungary
3 Department of Pharmaceutical Chemistry, Semmelweis University, Hőgyes Endre utca 9, H-1092 Budapest, Hungary
4 Integrative Health and Environmental Analysis Research Laboratory, Department of Analytical Chemistry, Institute of Chemistry, ELTE Eötvös Loránd University, Pázmány Péter sétány 1/A, H-1117 Budapest, Hungary
* Correspondence: fejos.ida@semmelweis.hu (I.F.); szabolcs.beni@ttk.elte.hu (S.B.)

Contents

HPLC measurements 2
CD measurements 4
CE measurements 6
NMR measurements 7
Following the synthetic procedures by NMR 14

HPLC measurements

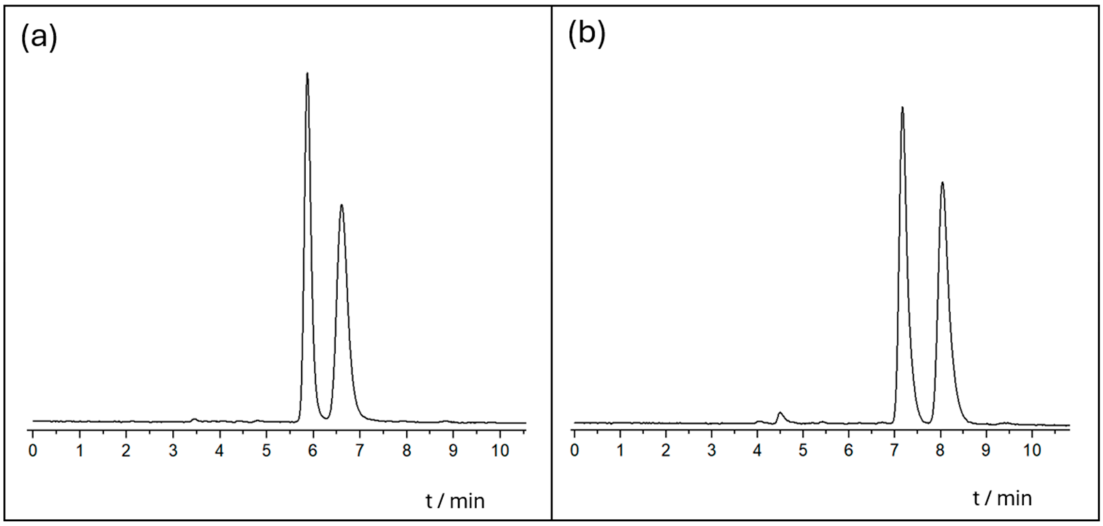

Figure S1. *(a)* HPLC chromatogram showing the separation of the enantiomers of Br-LAU using a **Chiralpak AD** column with methanol:diethylamine (MeOH:DEA) 100:0.1 as the eluent system. *(b)* HPLC chromatogram using a **Chiralcel OD** column under the same eluent conditions. Further conditions can be found in section *3.3. Chiral HPLC*.

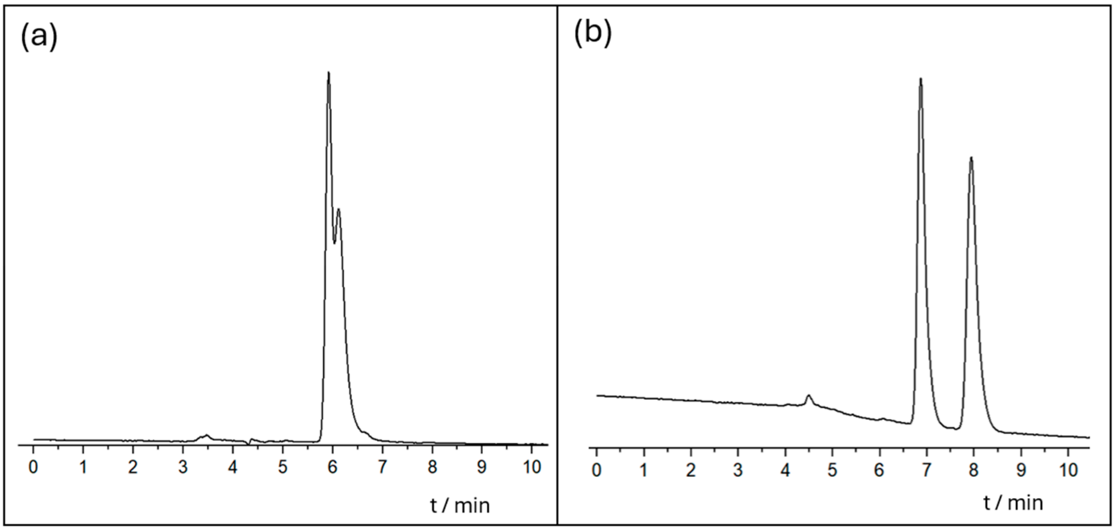

Figure S2. *(a)* HPLC chromatogram showing the partial separation of the enantiomers of LAU using a **Chiralpak AD** column with methanol:diethylamine (MeOH:DEA) 100:0.1 as the eluent system. *(b)* HPLC chromatogram using a **Chiralcel OD** column under the same eluent conditions. Further conditions can be found in section *3.3. Chiral HPLC*.

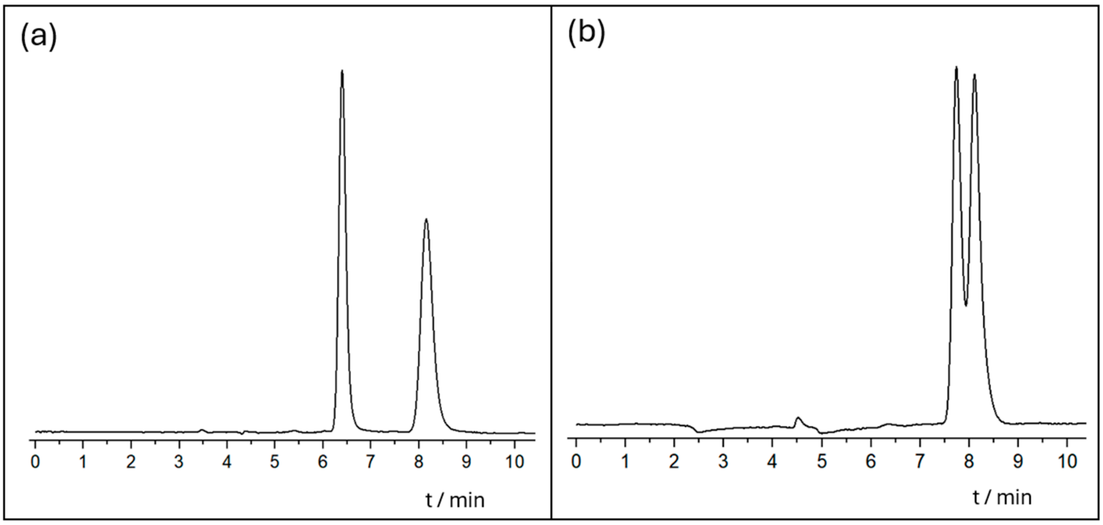

Figure S3. *(a)* HPLC chromatogram showing the separation of the enantiomers of NOR using a **Chiralpak AD** column with methanol:diethylamine (MeOH:DEA) 100:0.1 as the eluent system. *(b)* HPLC chromatogram using a **Chiralcel OD** column under the same eluent conditions. Further conditions can be found in section *3.3. Chiral HPLC*.

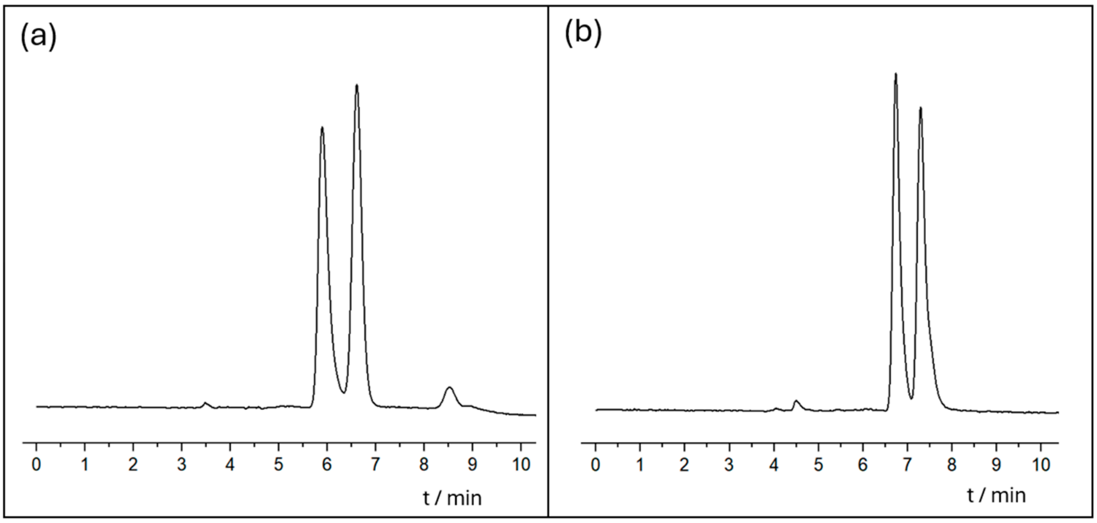

Figure S4. HPLC chromatograms showing the separation of the enantiomers of PROP using a *(a)* **Chiralpak AD** column and a *(b)* **Chiralcel OD** column with methanol:diethylamine (MeOH:DEA) 100:0.1 as the eluent system. Further conditions can be found in section *3.3. Chiral HPLC.*

CD measurements

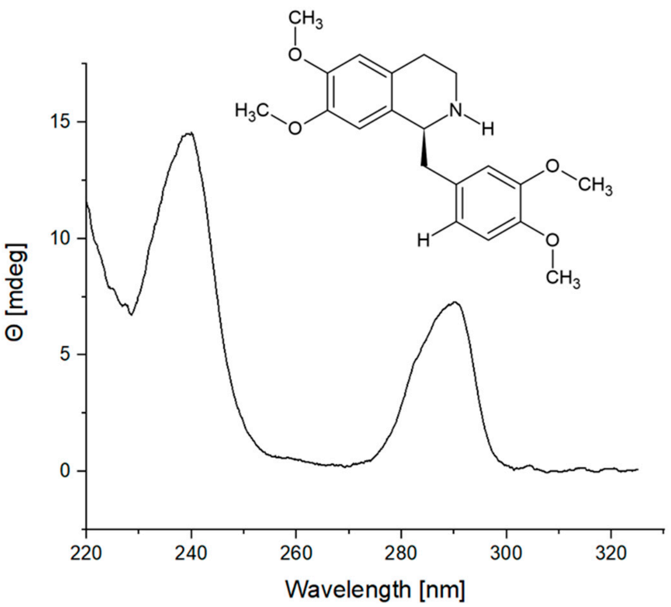

Figure S5. CD spectra of (*S*)-NOR. (0.3 mg/mL in MeOH). Further conditions can be found in Section *3.4. CD spectroscopy.*

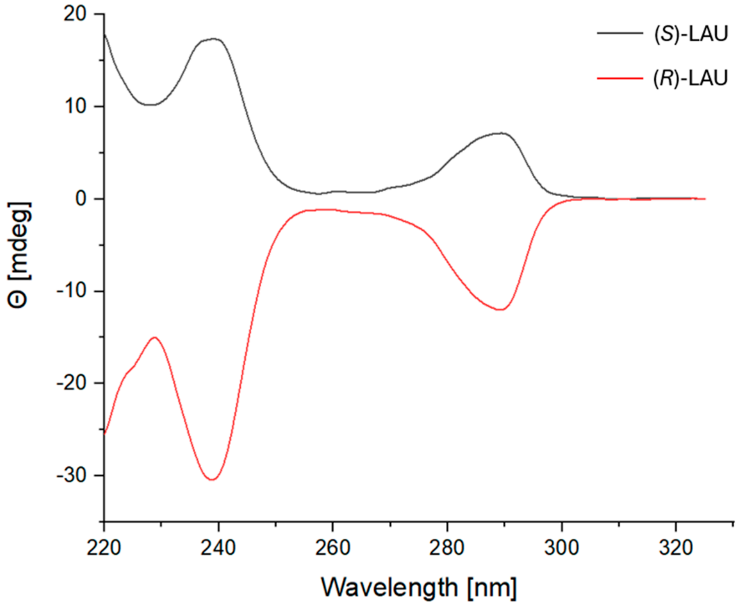

Figure S6. CD spectra of (*S*)-LAU (grey) and (*R*)-LAU (red). (0.3 and 0.5 mg/mL respectively in MeOH). Further conditions can be found in Section *3.4. CD spectroscopy.*

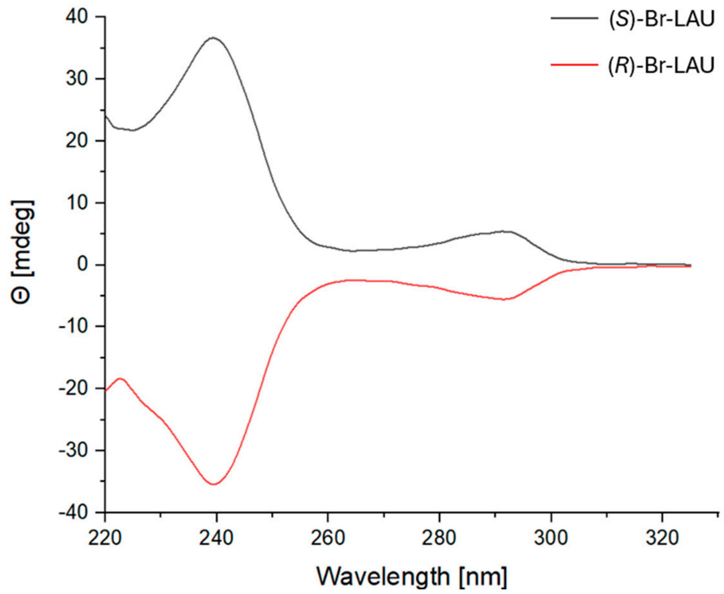

Figure S7. CD spectra of (*S*)-Br-LAU (grey) and (*R*)-Br-LAU (red). (0.3 mg/mL in MeOH). Further conditions can be found in Section *3.4. CD spectroscopy*.

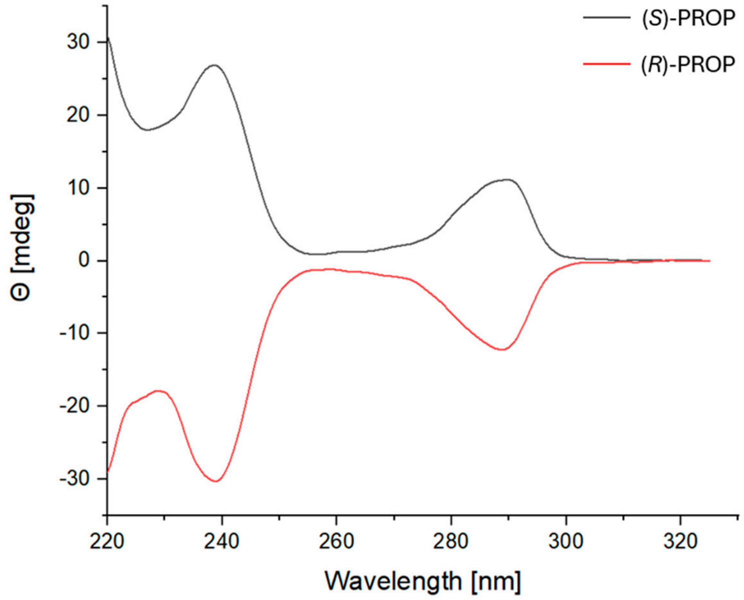

Figure S8. CD spectra of (*S*)-PROP (grey) and (*R*)-PROP (red). (0.3 mg/mL in MeOH). Further conditions can be found in Section *3.4. CD spectroscopy*.

CE measurements

Table S1. Some averaged apparent LAU-CyD complex stability constants (***K****stab,* / M^{-1}) and complex mobilities ($\mu_{(AS)}$ / 10^{-5} $cm^2V^{-1}s^{-1}$) of the transient diastereomeric complexes measured by affinity capillary electrophoresis at 30 mM phosphate buffer (pH 7.4), 25 °C, 15 kV, 200 nm. Further conditions and CyD abbreviations can be found in Section *3.5. Capillary Electrophoresis, 3.1. Materials.*

		**first migrating enantiomer**	**second migrating enantiomer**
S-β-CyD	$\mu_{(AS)}$	-6.6 ± 0.7	-8.0 ± 1.1
	K_{stab}	660 ± 65	660 ± 92
SBE-β-CyD	$\mu_{(AS)}$	-11.1 ± 0.7	-12.0 ± 0.7
	K_{stab}	476 ± 43	476 ± 44
SP-β-CyD	$\mu_{(AS)}$	1.4 ± 0.8	1.4 ± 0.8
	K_{stab}	131 ± 18	131 ± 18
CM-β-CyD	$\mu_{(AS)}$	1.0 ± 1.4	1.7 ± 1.7
	K_{stab}	161 ± 41	185 ± 56
CM-γ-CyD	$\mu_{(AS)}$	3.1 ± 1.0	1.2 ± 0.6
	K_{stab}	155 ± 45	155 ± 19
SBX	$\mu_{(AS)}$	-19.0 ± 1.5	-20.5 ± 1.5
	K_{stab}	210 ± 21	205 ± 20
SGX	$\mu_{(AS)}$	-33.6 ± 0.3	-33.0 ± 0.2
	K_{stab}	2 070 ± 55	3 590 ± 82

Table S2. Averaged apparent alkaloid–CyD complex stability constants (***K****stab,,* / M^{-1}) and complex mobilities ($\mu_{(AS)}$ / 10^{-5} $cm^2V^{-1}s^{-1}$) measured by affinity capillary electrophoresis at 30 mM phosphate buffer (pH 7.4), 25 °C, 15 kV, 200 nm. Further conditions and CyD abbreviations can be found in Section *3.5. Capillary Electrophoresis, 3.1. Materials.*

		**(*S*)-LAU**	**(*R*)-LAU**	**(*S*)-Br-LAU**	**(*R*)-Br-LAU**	**(*S*)-PROP**	**(*R*)-PROP**	**(*S*)-NOR**	**(*R*)-NOR**
SBX	$\mu_{(AS)}$	-19.0 ±1.5	-20.5 ± 1.5	-19.4 ± 1.0	-19.2 ± 1.0	-25.1 ± 4.7	-25.1 ± 4.7	-21.1 ± 0.7	-21.4 ± 0.8
	K_{stab}	210 ± 21	205 ± 20	193 ± 14	167 ± 11	278 ± 80	278 ± 80	340 ± 17	387 ± 25
SGX	$\mu_{(AS)}$	-33.6 ± 0.3	-33.0 ± 0.2	-35.9 ± 4.3	-38.9 ± 5.7	-34.0 ± 0.8	-33.4 ± 0.5	-34.2 ± 0.4	-33.5 ± 0.3
	K_{stab}	2070 ± 55	3 590 ± 82	680 ± 126	603 ± 136	2 561 ± 150	3 812 ± 173	3 000 ± 94	4 620 ± 120

NMR measurements

Table S3. Complete ^{1}H NMR resonances assignment for LAU in D_2O (30 mM phosphate buffer pD 7.4 ; 298 K; 500 MHz).

Atom position	^{1}H δ (ppm)
1	-
2a	3.69 (1H, m)
2b	3.35 (1H, m)
3a	3.00 (1H, m)
3b	3.10 (1H, m)
4	-
5	6.86 (1H, s)
6	-
7	-
8	5.82 (1H, s)
9	-
10	4.48 (1H, dd, broad)
11a	2.96 (1H, m)
11b	3.38 (1H, m)
12	-
13	6.48 (1H, d, J=1.5 Hz)
14	-
15	-
16	6.93 (1H, d, J=8.3 Hz)
17	-
18	6.72 (1H, dd, J=8.3 Hz, 1.5 Hz)
19	-
20	3.76 (3H, s)
21	-
22	3.35 (3H, s)
23	-
24	3.59 (3H, s)
25	-
26	3.76 (3H, s)
27	2.90 (3H, s)

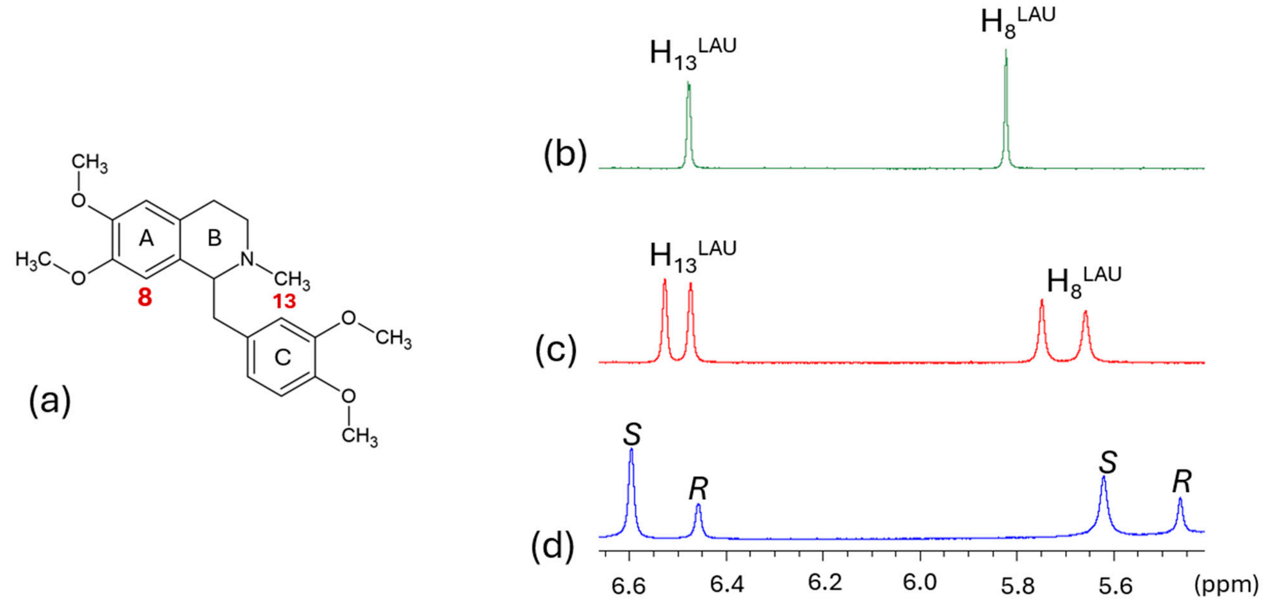

Figure S9. (a) H8 and H13 aromatic protons of LAU. (b) LAU in 30 mM phosphate buffer in D_2O pD 7.4. (c) SGX:LAU=0.38:1 complex and (d) spiked LAU-SGX=1:1 complex in the same buffer system. The samples were spiked with (S)-enantiomers, 30 mM phosphate buffer in D_2O pD 7.4 solutions containing 10 mM of the alkaloids and 10 mM SGX.

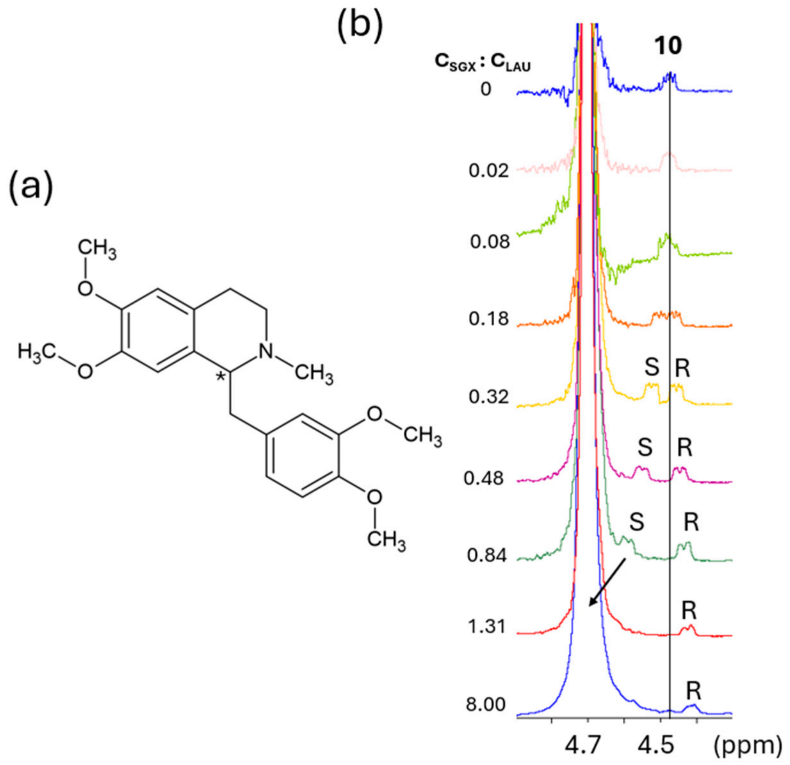

Figure S10. (a) The structure of LAU, labelled its chiral center (H10 proton). (b) Part of ^{1}H NMR spectra of LAU in pD 7.4 phosphate buffer ^{1}H titration recorded at 500 MHz, denoted the H10 protons of (*S*)- and (*R*)-LAU.

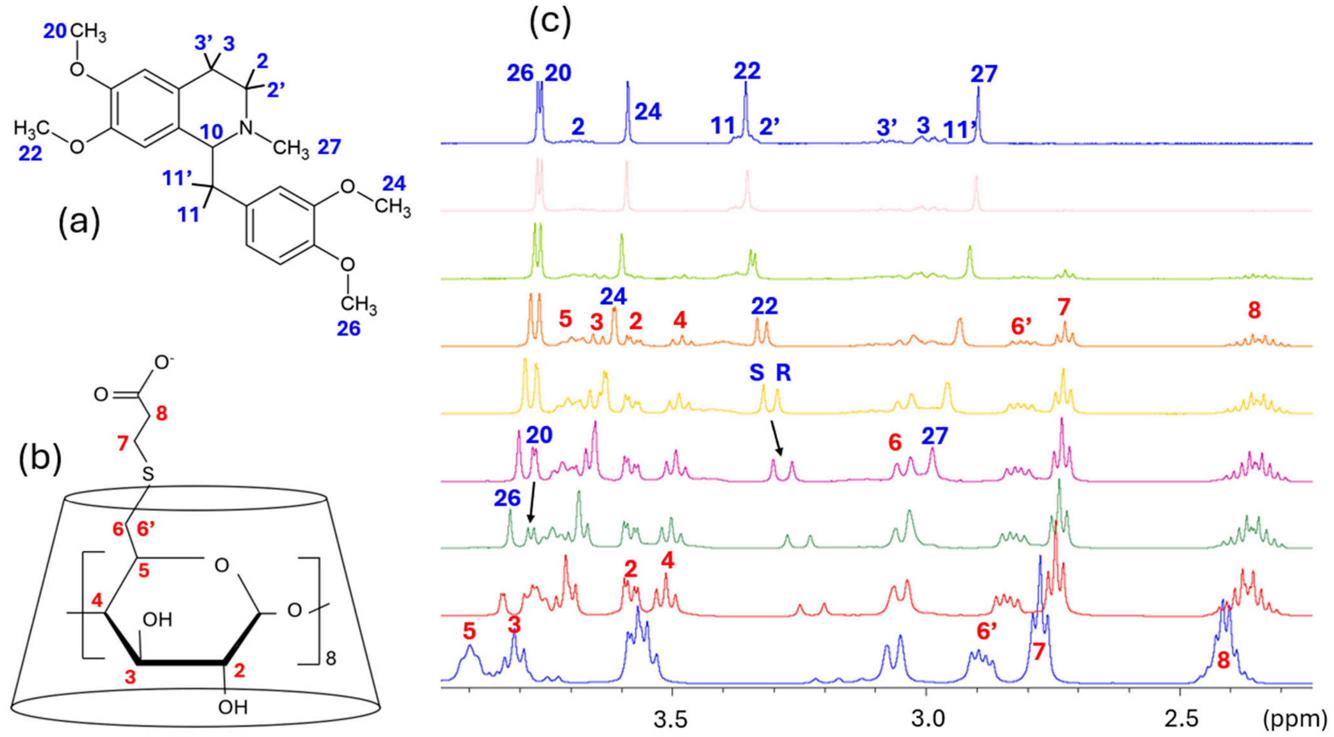

Figure S11. (a) The structure of LAU, labelled its aliphatic protons. (b) The structure of SGX, numbered its H2-H8 protons. Part of ^{1}H NMR spectra of LAU in pD 7.4 phosphate buffer ^{1}H titration recorded at 500 MHz, denoted the protons of LAU (blue), SGX (red).

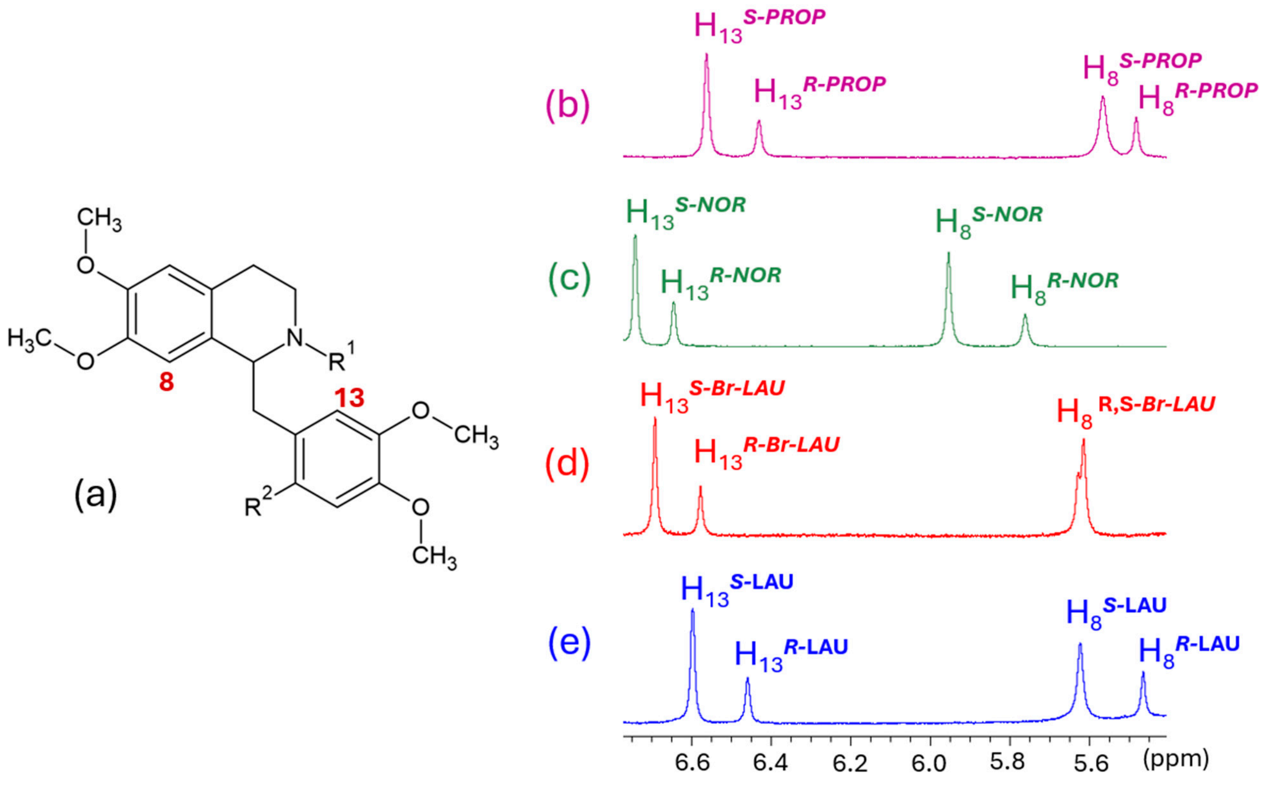

Figure S12. (a) H8 and H13 aromatic protons of LAU derivatives. (R^1=R^2= H NOR, R^1= CH_3 R^2= H LAU, R^1=CH_3 R^2= Br Br-LAU, R^1=$CH_2CH_2CH_3$ R^2= H PROP), Part of the ^{1}H NMR spectra (aromatic region) of alkaloid-SGX complexes; (b) PROP-SGX=1:1, (c) NOR-SGX=2:1, (d) Br-LAU-SGX=1:2, (e) LAU-SGX=1:1.

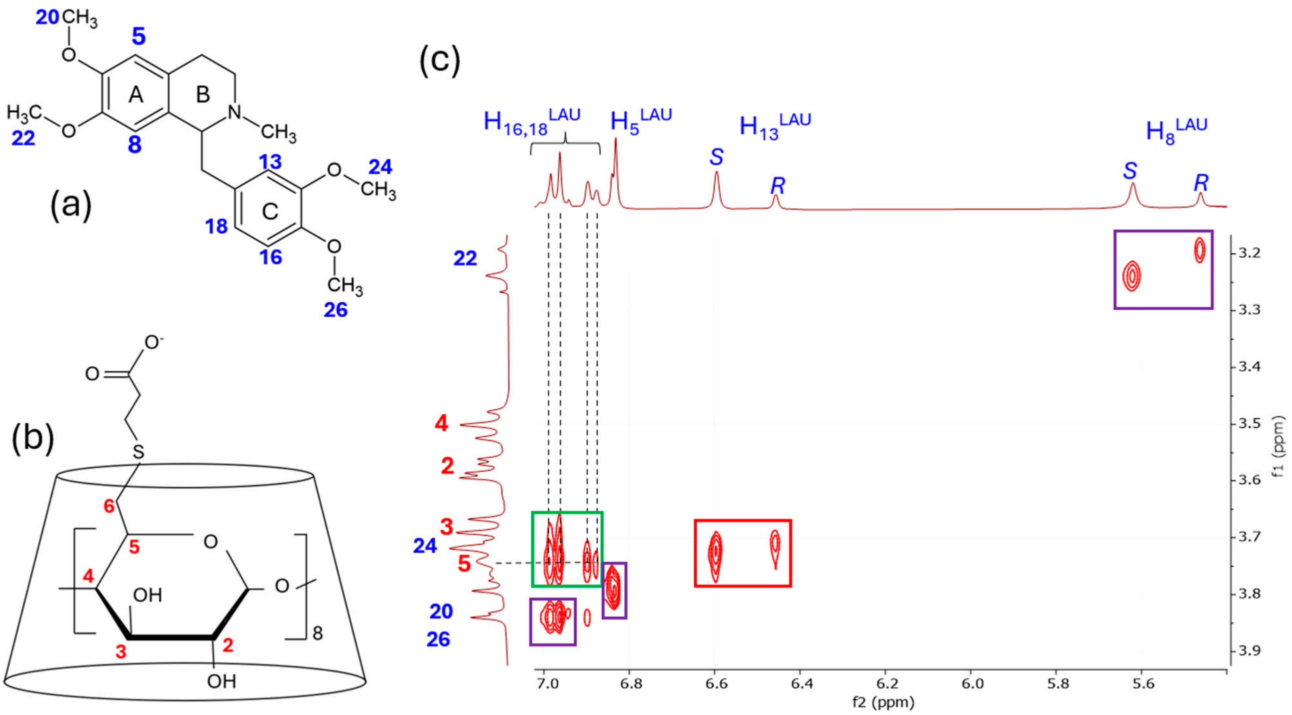

Figure S13. (a) The structure of LAU, labelled H5, H8, H13, H20, H22, H24 and H26 protons of them. (b) Schematic representation of SGX and its H2, H3, H4, H5 and H6 protons. (c) Partial 2D ROESY NMR spectrum of the LAU-SGX (1:1) complex. The intramolecular interaction between H16, H18 and H5 protons of SGX (in the green box). The intermolecular interaction between aromatic and methoxy signals of LAU (in the purple box). Intermolecular and intramolecular interactions might overlap (in the red box). The sample was spiked with (*S*)-LAU, 30 mM phosphate buffer in D_2O pD 7.4 solution, recorded at 400 MHz.

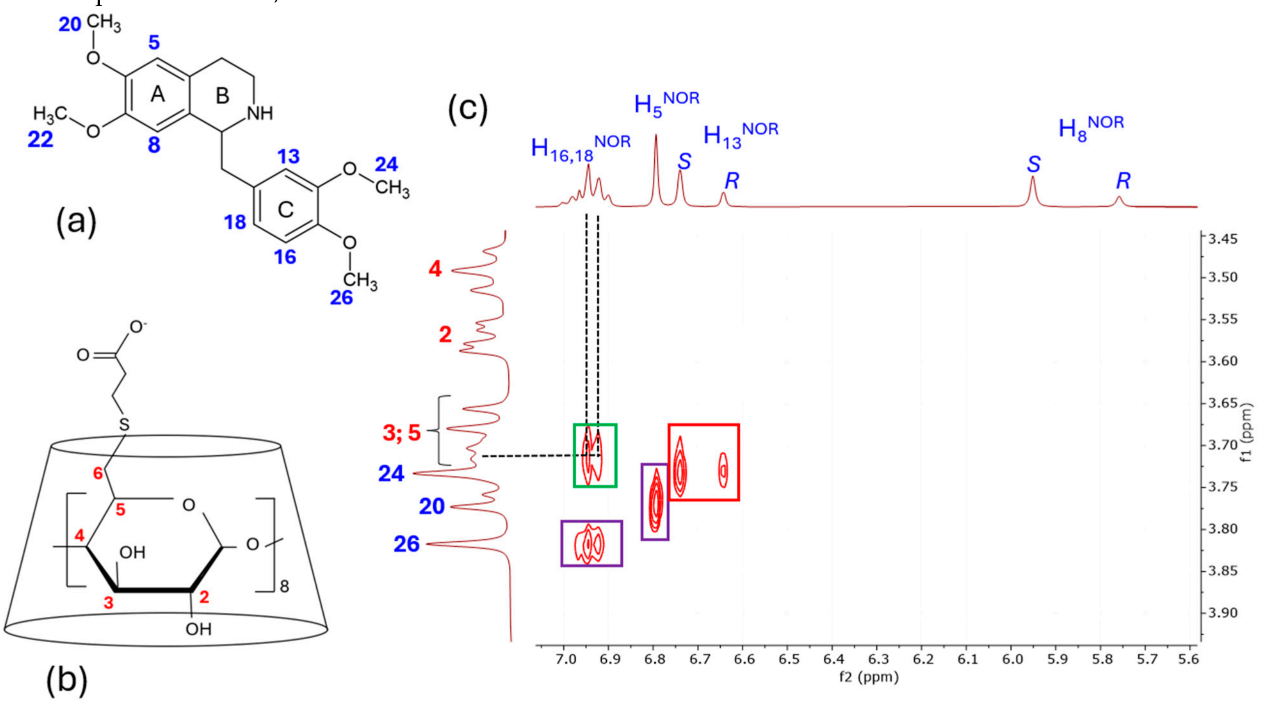

Figure S14. (a) The structure of NOR, labelled H5, H8, H13, H20, H22, H24 and H26 protons of them. (b) H2, H3, H4 and H5 protons of SGX. (c) Partial 2D ROESY NMR spectrum of the NOR-SGX (2:1) complex, recorded at 400 MHz. The intermolecular interactions between aromatic and methoxy signals of NOR (in the purple box). The intramolecular interactions between SGX H3, H5 and H16, H18 signals of NOR (in the green box). Intermolecular and intramolecular interactions might overlap (in the red box). The sample was spiked with (*S*)-NOR, 30 mM phosphate buffer in D_2O pD 7.4 solution.

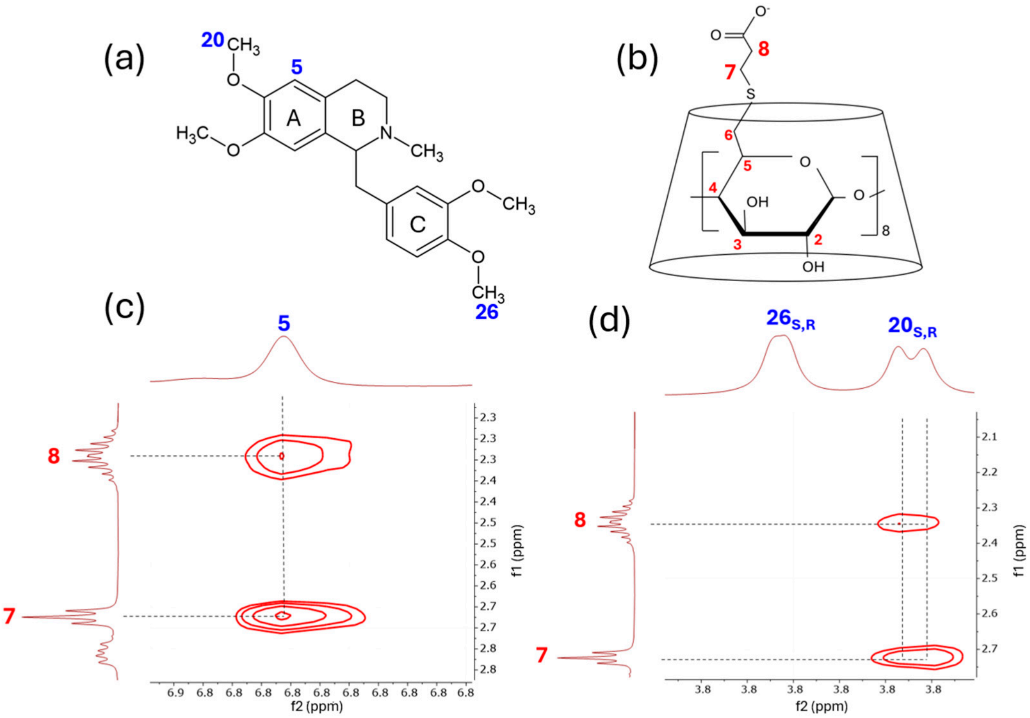

Figure S15. (a) Chemical structure and atom numbering of LAU, along with (b) the schematic representation and numbering of SGX. (c) Partial 2D ROESY NMR spectrum of the LAU-SGX (1:1) complex. The intramolecular interaction between H7, H8 protons of SGX and H5 proton of LAU. (d) Partial 2D ROESY NMR spectrum of the LAU-SGX (1:1) complex. The intramolecular interaction between H7, H8 protons of SGX and H20 proton of (*S*)- and (*R*)-LAU. Further conditions can be found in section 3.6. NMR spectroscopy.

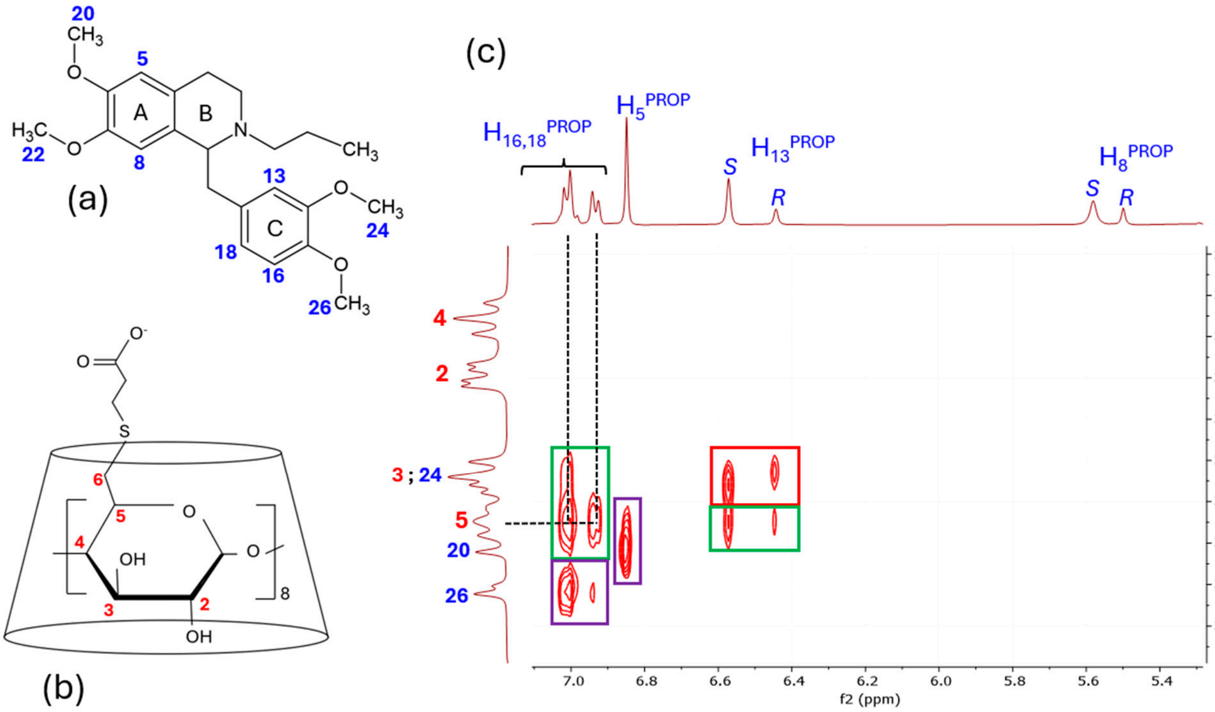

Figure S16. (a) The structure of PROP, labelled H5, H8, H13, H16, H18 and H22 protons of PROP. (b) H2, H3, H4 and H5 protons of SGX. (c) Partial 2D ROESY NMR spectrum of the PROP-SGX complex, recorded at 500 MHz. The intramolecular interactions between H8 aromatic and H22 methoxy signals of PROP (in the purple box). The intermolecular interactions between SGX H3, H5 and H5, H8, H13, H16, H18 aromatic signals of PROP (in the green box and marked with dotted lines). Intermolecular and intramolecular interactions might overlap (in the red box). The sample was spiked with (*S*)-PROP, 30 mM phosphate buffer in D_2O pD 7.4 solution.

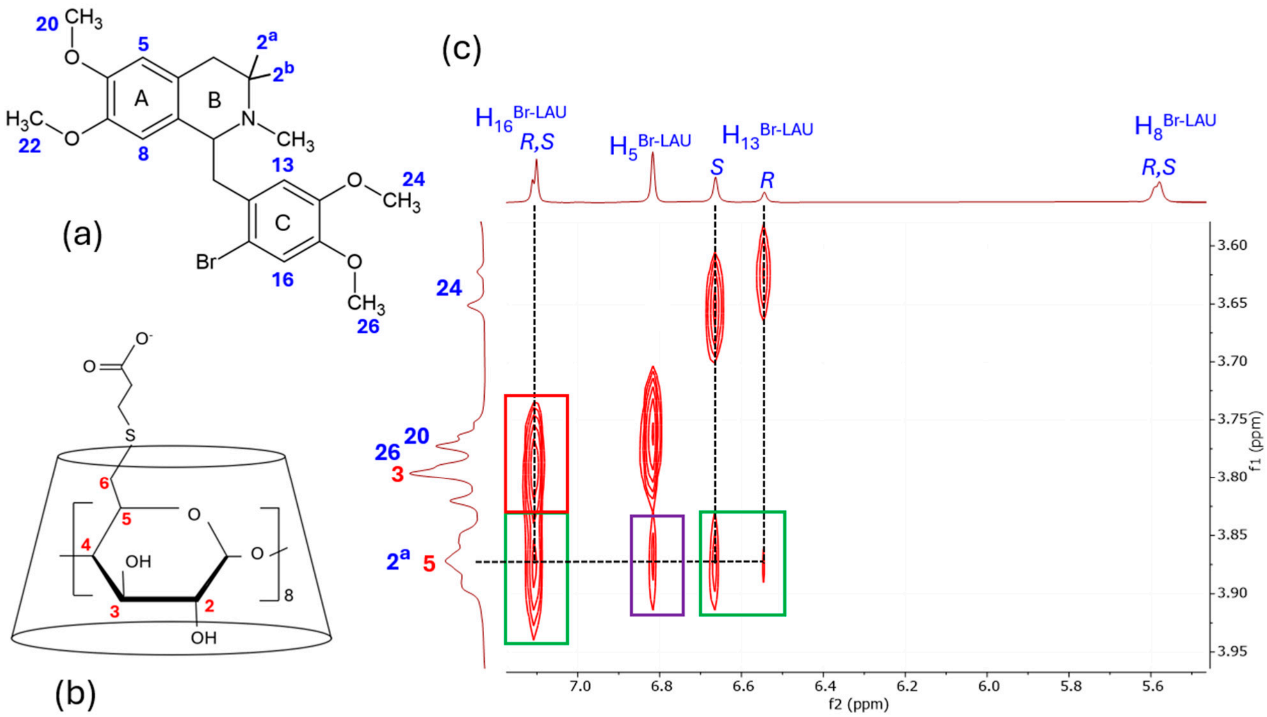

Figure S17. (a) The structure of Br-LAU and its H5, H8, H13 and H16 aromatic protons labelled with blue. (b) Schematic representation of SGX and its H2, H3, H4, H5 and H6 protons. (c) Partial 2D ROESY NMR spectrum of the Br-LAU-SGX (1 : 2.5) complex, recorded at 400 MHz, denoted the spatial proximity between SGX the H5 and the H13, H16 aromatic signals of Br-LAU (in the green box). Intramolecular interactions between H2a signal and H5 aromatic protons of Br-LAU (in the purple box). Intermolecular and intramolecular interactions may overlap (in the red box). The sample was spiked with (S)-Br-LAU, 30 mM phosphate buffer in D_2O pD 7.4 solution.

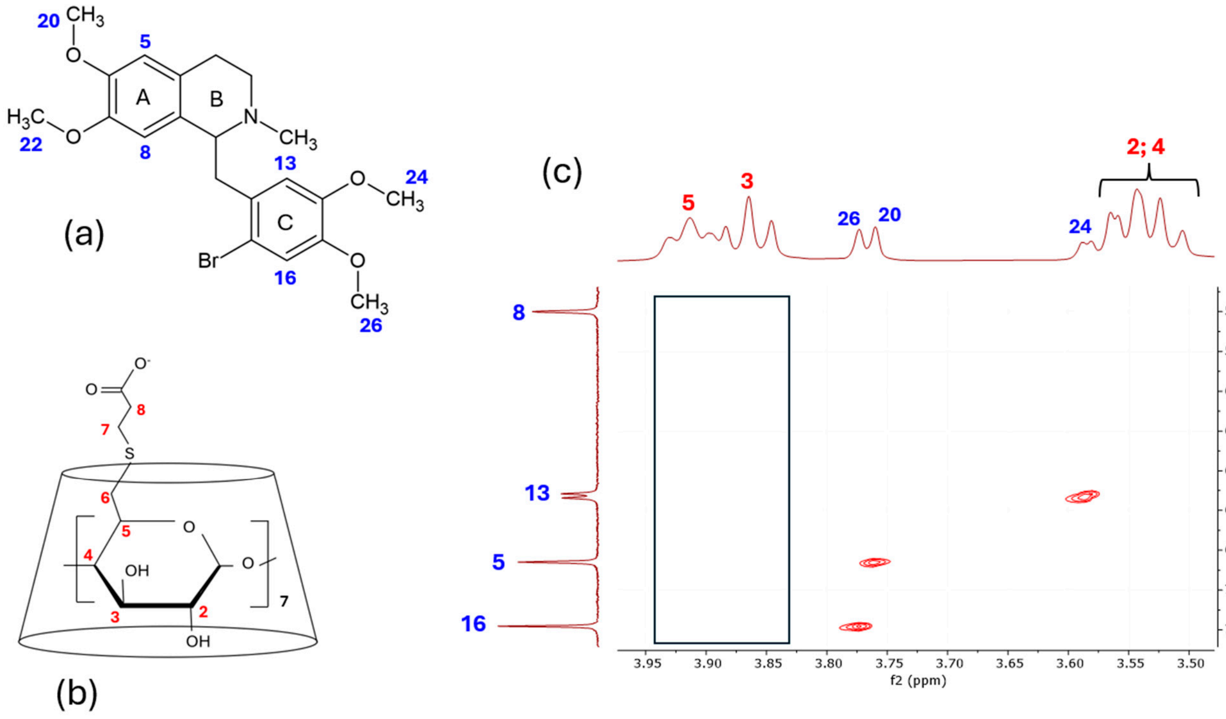

Figure S18. Partial 2D ROESY NMR spectrum of Br-LAU-SBX (1 : 2) complex, 30 mM phosphate buffer in D_2O pD 7.4 solution.

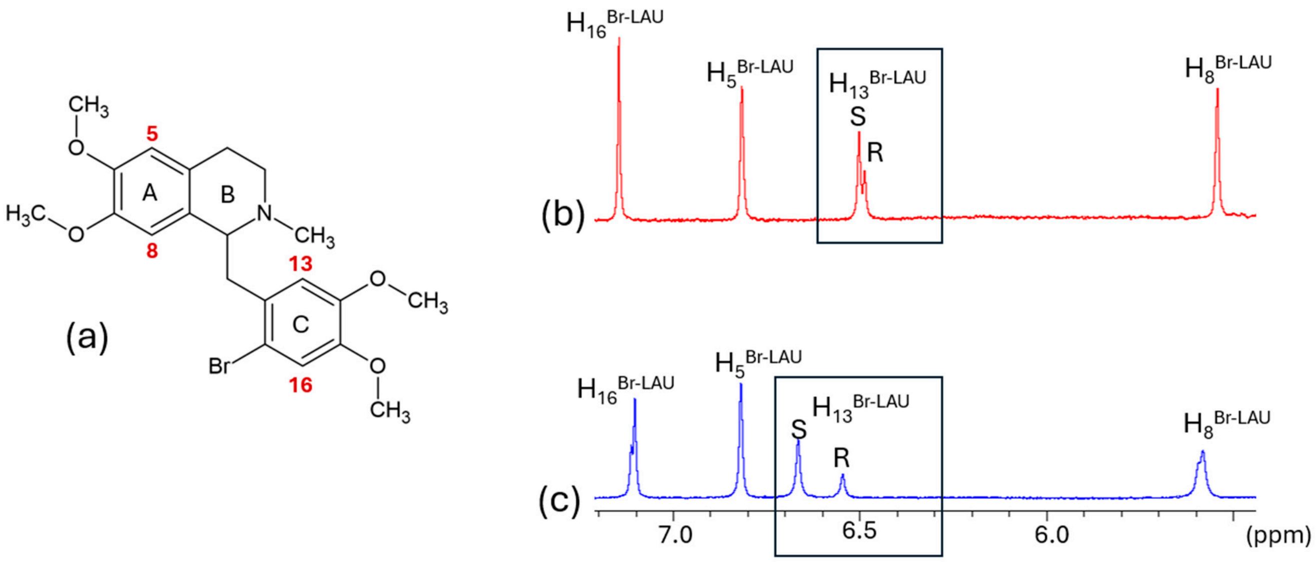

Figure S19. (a) The structure of Br-LAU and its H5, H8, H13 and H16 aromatic protons labelled with brown. ^{1}H spectra of Br-LAU SBX complex and Br-LAU SGX complex. The sample was spiked with (S)-Br-LAU, 30 mM phosphate buffer in D_2O pD 7.4 solution.

Following the synthetic procedures by NMR

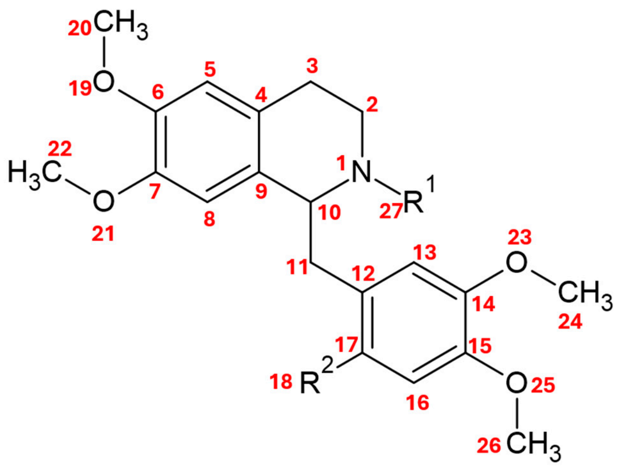

Figure S20. The basic structure of LAU derivatives. R_1=CH_3 and R_2=H Laudanosine (LAU), R_1=R_2=H Norlaudanosine (NOR), R_1=$CH_2CH_2CH_3$ and R_2=H N-propyl-norlaudanosine (PROP), R_1=CH_3 and R_2=Br 6′-Br-laudanosine (Br-LAU).

Table S4. ^{1}H and ^{13}C assignment of LAU derivatives, (in $CDCl_3$-d, 400 MHz). For the atomic positions of LAU derivatives see Figure S9.

	LAU		NOR		PROP		Br-LAU	
**Atomic position**	**^{1}H δ (ppm)**	**^{13}C δ (ppm)**	**^{1}H δ (ppm)**	**^{13}C δ (ppm)**	**^{1}H δ (ppm)**	**^{13}C δ (ppm)**	**^{1}H δ (ppm)**	**^{13}C δ (ppm)**
1	-	-	-	-	-	-	-	-
2	2.77 - 3.18 (2H, m)	46.9	3.14 -3.28 (2H, m)	38.6	3.52-3.73 (2H, m)	42.2	2.73 - 3.18 (2H, m)	46.5
3	2.60-2.84 (2H, m)	25.5	2.83-3.10 (2H, m)	25.1	3.05 (2H, m)	21.4	2.56 -2.83 (2H, m)	25.3
4	-	125.9	-	123.7	-	120.8	-	126.1
5	6.57 (1H, s)	111.2	6.50 (1H, s)	111.2	6.66 (1H, s)	111.1	6.51 (1H, s)	111.3
6	-	147.3	-	148.7	-	149.3	-	147.4
7	-	146.3	-	147.5	-	147.3	-	146.3
8	6.08 (1H, s)	111.1	6.06 (1H, s)	110.0	5.73 (1H, s)	111.2	5.96 (1H, s)	111.3
9	-	129.1	-	122.9	-	121.1	-	128.9
10	3.71 (1H, dd, J= 7.8, 4.9 Hz)	64.9	4.68 (1H, dd, J= 6.8, 3.3 Hz)	55.0	4.31 (1H, dd, broad)	64.6	3.73 (1H, dd, broad)	62.7

11	2.79 (1H, m, overlap) and 3.16 (1H, m, overlap)	40.9	3.12 (1H, m, overlap) and 3.52 (1H, m, overlap)	40.6	2.95 (1H, m, overlap) and 4.20 (1H, dd, J=12.7, 2.6 Hz)	41.1	2.81 (1H, m, overlap) and 3.16 (1H, m, overlap)	40.5
12	-	132.5	-	128.0	-	128.0	-	131.2
13	6.62 (1H, d, J=1.8 Hz)	113.0	6.71 (1H, d, overlap)	113.2	6.78 (1H, d, J=1.8 Hz)	113.3	6.45 (1H, s)	115.1
14	-	148.6	-	149.0	-	149.0	-	148.0
15	-	147.4	-	148.3	-	148.3	-	147.8
16	6.78 (1H, d, J=8.1 Hz)	111.0	6.70 (1H, d, J=9.0 Hz)	111.2	6.74 (1H, d, J=8.2 Hz)	111.0	6.94 (1H, s)	115.3
17	-	121.9	-	122.4	-	122.7	-	114.9
18	6.67 (1H, dd, J=8.1 Hz, 1.8 Hz)	-	6.64 67 (1H, dd, broad)	-	6.56 (1H, dd, J=8.2 Hz, 1.8 Hz)	-	-	-
19	-	-	-	-	-	-	-	-
20	3.86 (3H, s)	55.8	3.77 (3H, s)	55.9	3.87 (3H, s)	55.96	3.77 (3H, s)	55.8
21	-	-	-	-	-	-	-	-
22	3.60 (3H, s)	55.6	3.50 (3H, s)	55.7	3.48 (3H, s)	54.4	3.50 (3H, s)	55.5
23	-	-	-	-	-	-	-	-
24	3.86 (3H, s)	55.8	3.72 (3H, s)	56.0	3.81 (3H, s)	56.2	3.78 (3H, s)	56.2
25	-	-	-	-	-	-	-	-
26	3.87 (3H, s)	55.9	3.77 (3H, s)	55.9	3.84 (3H, s)	55.94	3.64 (3H, s)	56.0
27	2.56 (3H, s)	42.7	1.79 (1H, s broad)	-			2.47 (3H, s)	42.7

Table S5. ^{1}H and ^{13}C chemical shifts of propyl group of PROP (in $CDCl_3$-d, 400 MHz).

**Atom position**	**^{1}H δ (ppm)**	**^{13}C δ (ppm)**
1′ (CH_2)	3.00-3.11 (2H, m)	54.4
2′(CH_2)	2.04- 2.20 (2H, m)	18.1
3′(CH_3)	0.97 (3H, t)	11.2

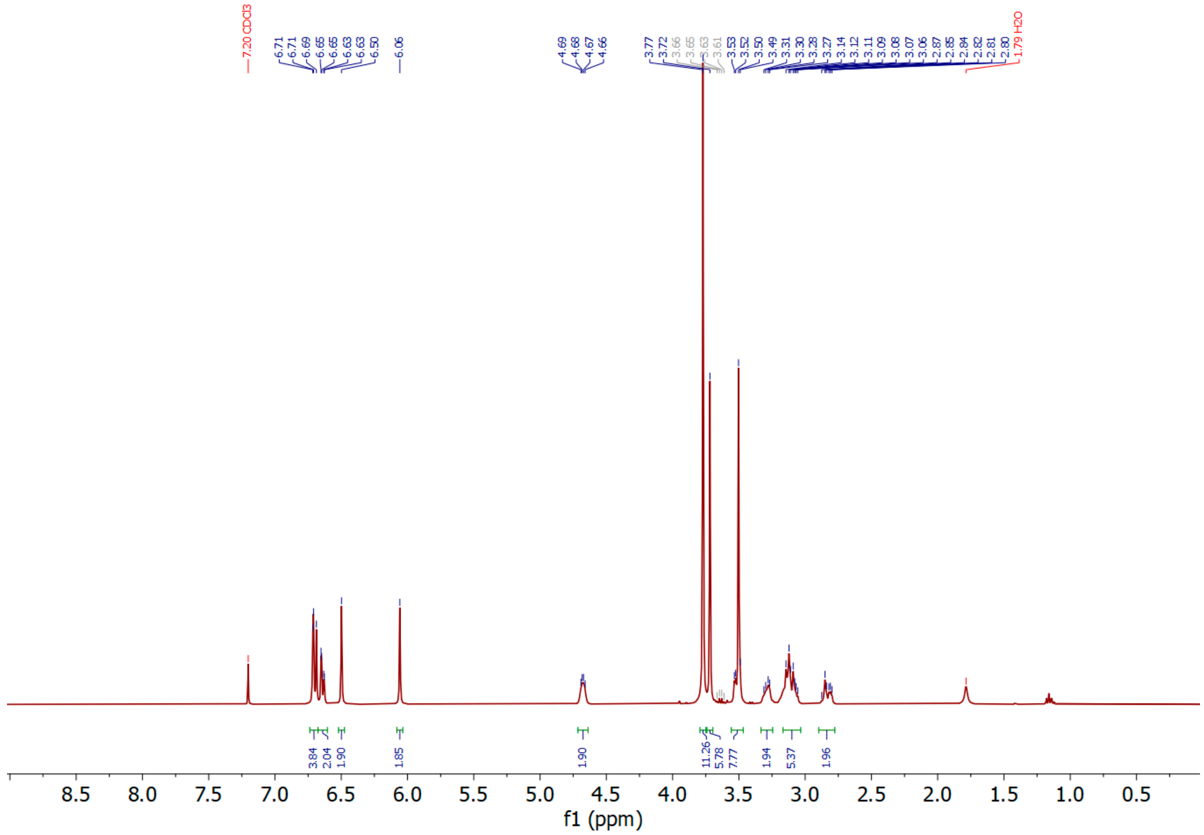

Figure S21. ^{1}H NMR spectrum of racemic NOR. (in $CDCl_3$, 298 K, 400 MHz).

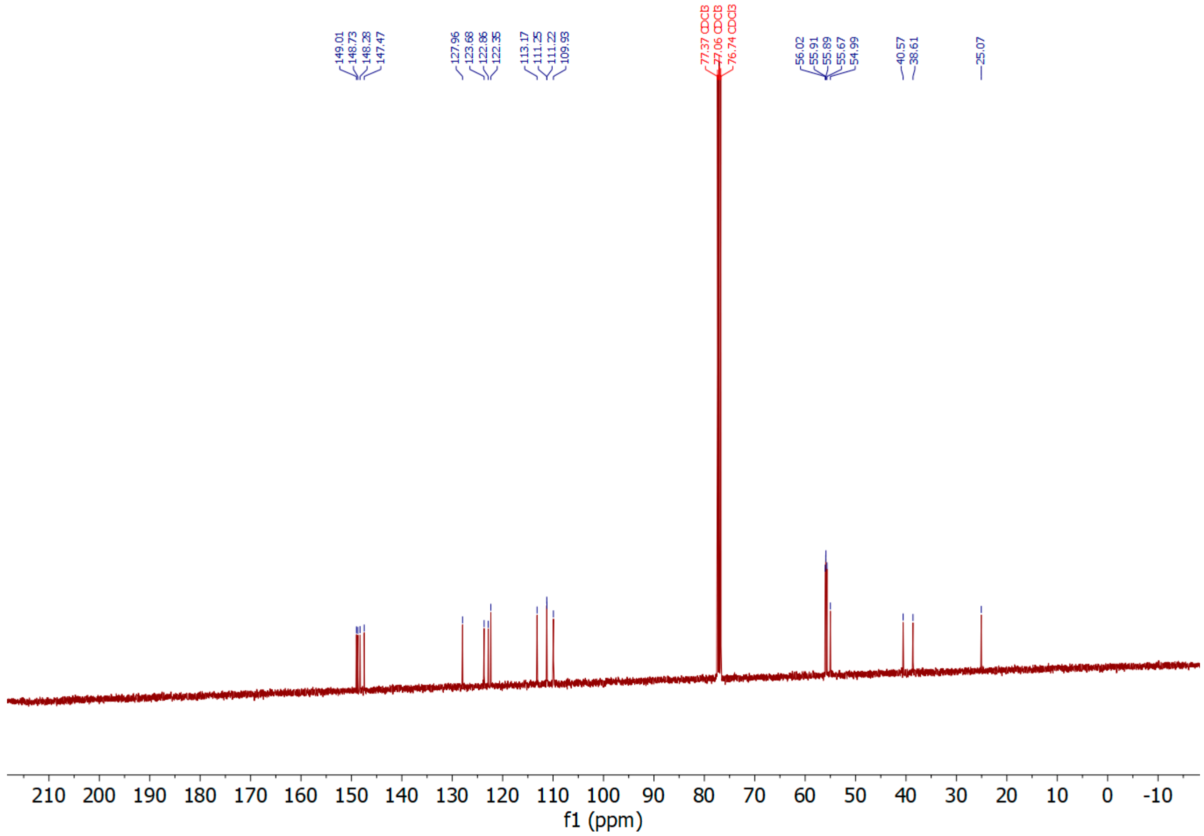

Figure S22. ^{13}C NMR spectrum of racemic NOR. (in $CDCl_3$, 298 K, 400 MHz).

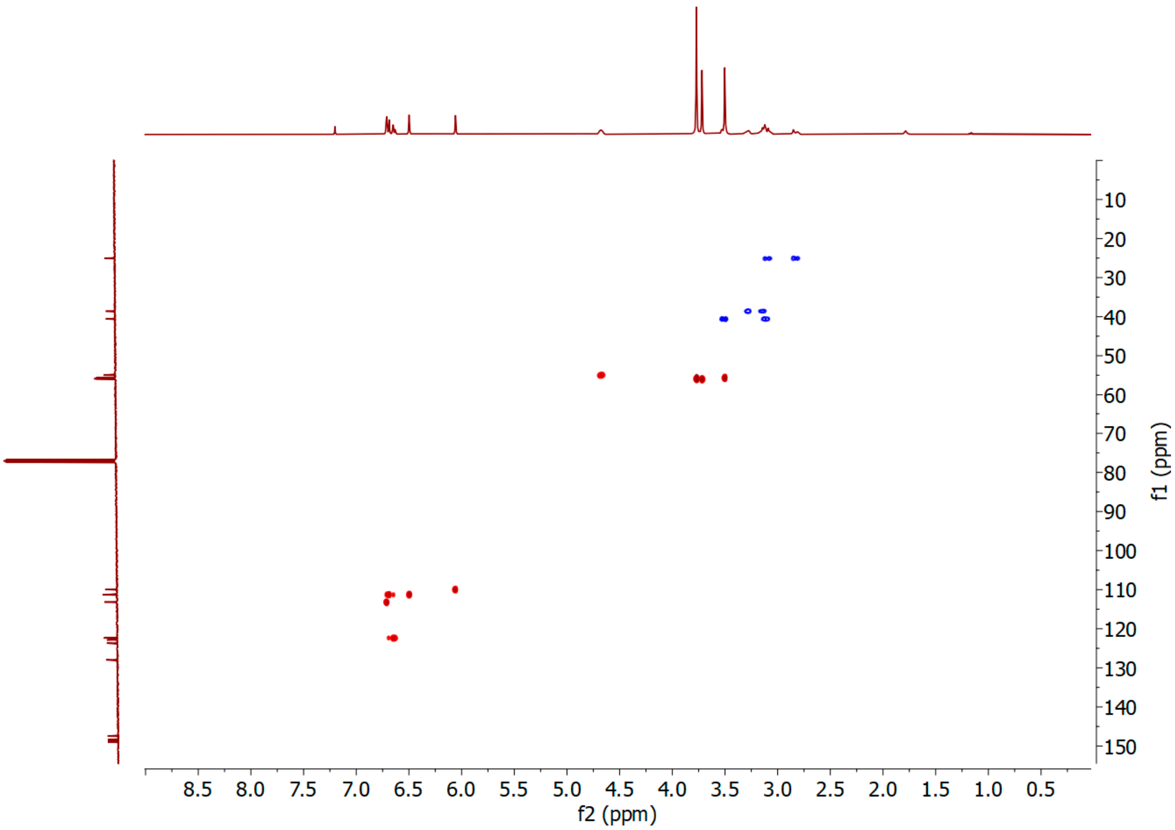

Figure S23. HSQC spectrum of racemic NOR. (in $CDCl_3$, 298 K, 400 MHz).

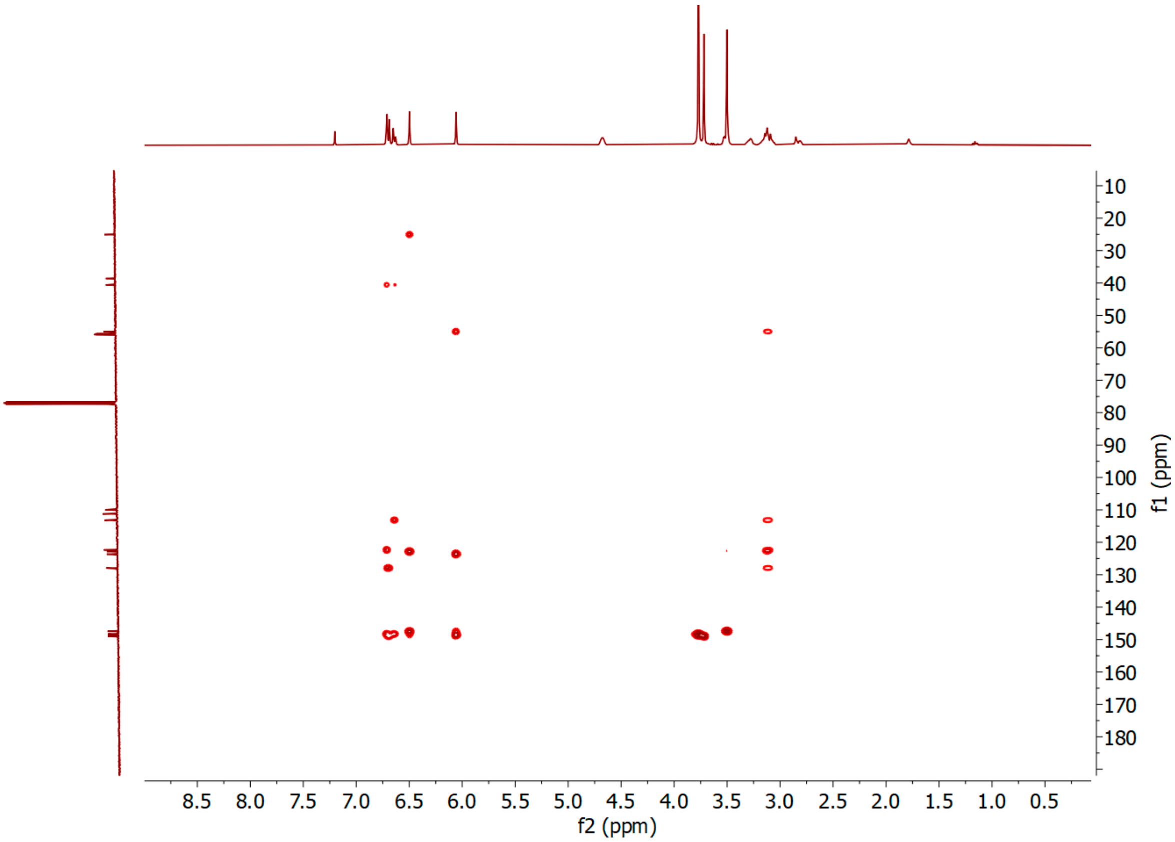

Figure S24. HMBC spectrum of racemic NOR. (in $CDCl_3$, 298 K, 400 MHz).

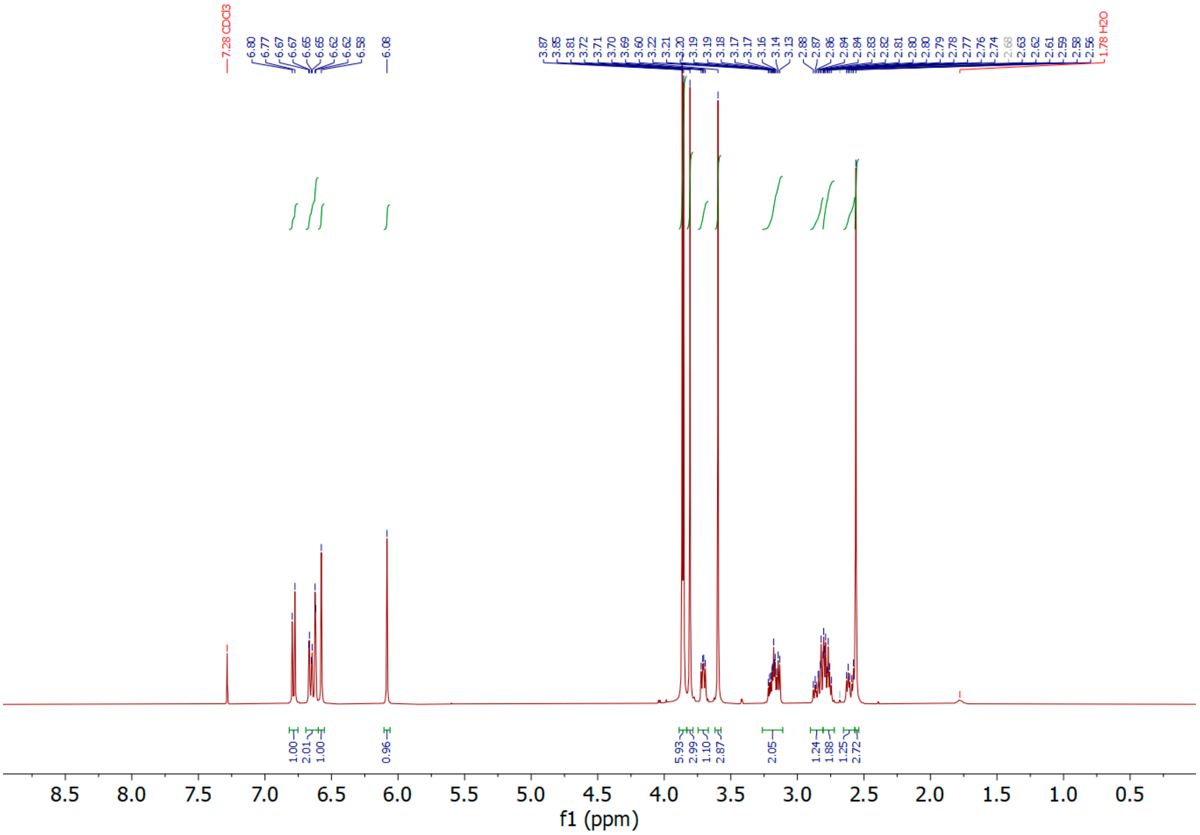

Figure S25. ^{1}H NMR spectrum of racemic LAU. (in $CDCl_3$, 298 K, 400 MHz).

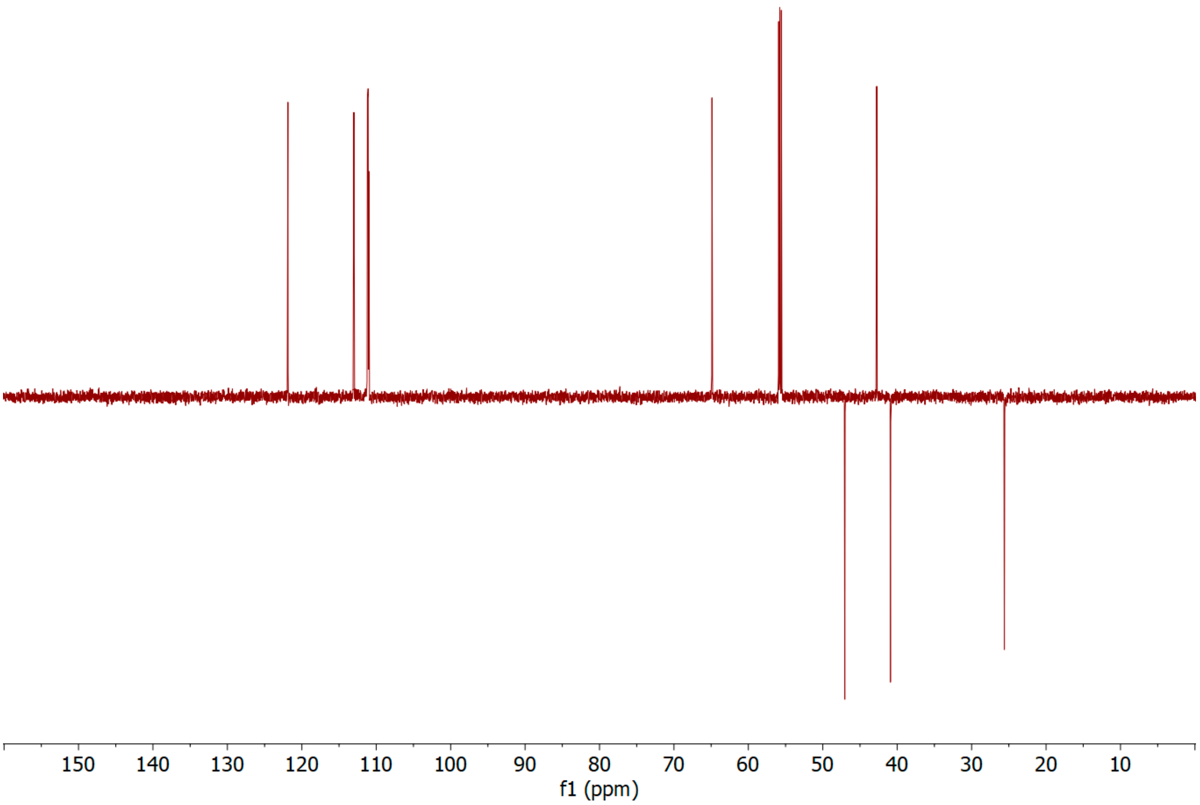

Figure S26. ^{13}C DEPT NMR spectrum of racemic LAU. (in $CDCl_3$, 298 K, 400 MHz).

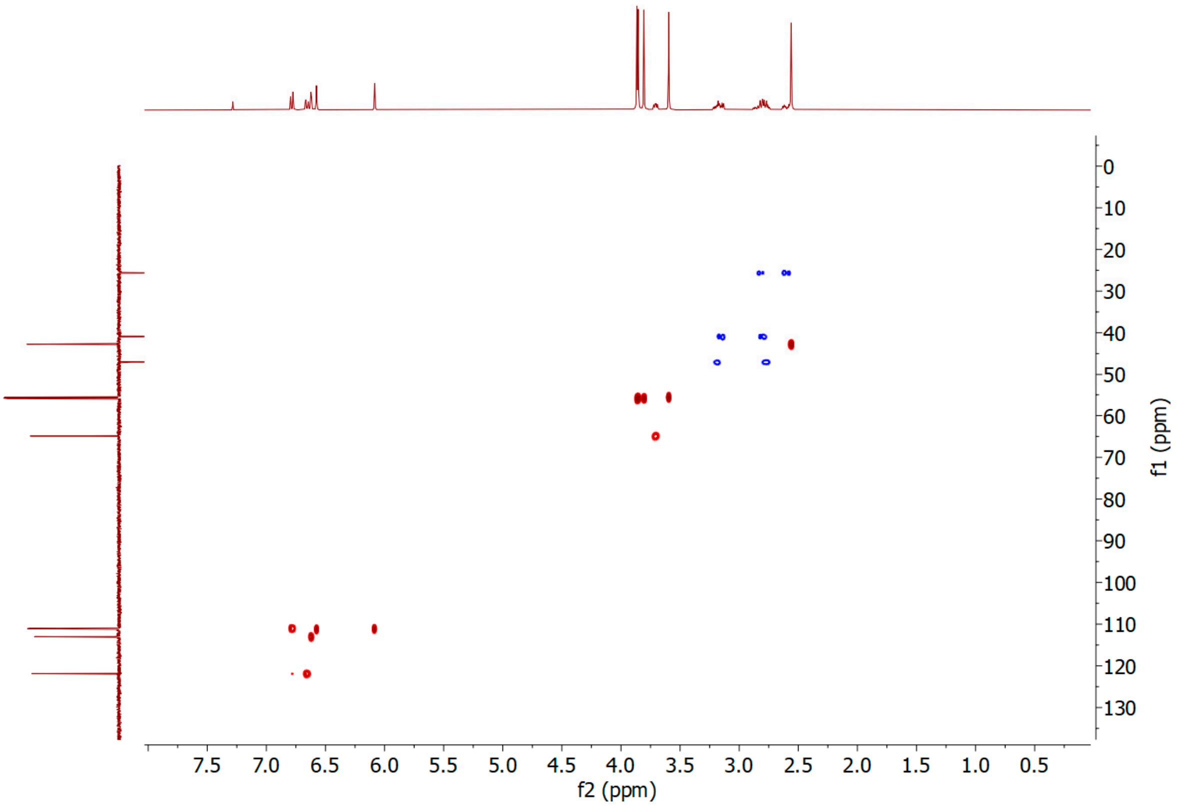

Figure S27. HSQC spectrum of racemic LAU. (in $CDCl_3$, 298 K, 400 MHz).

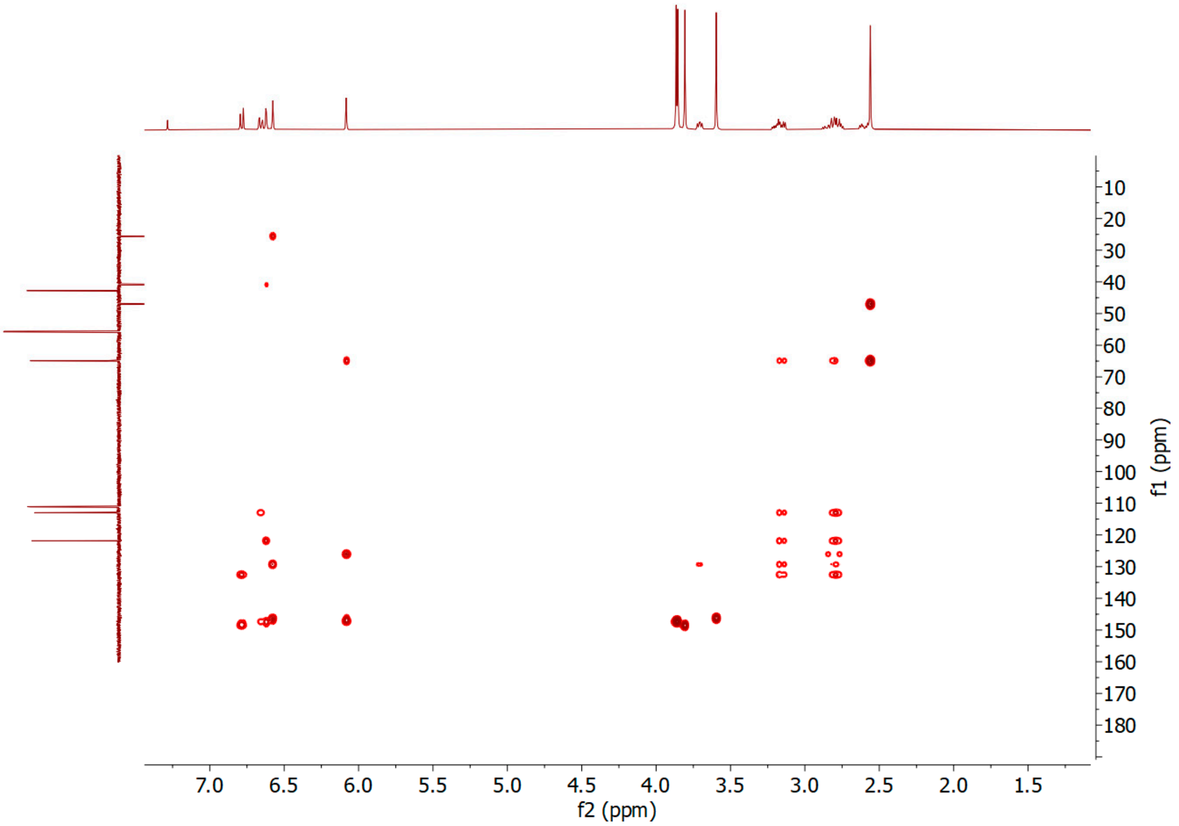

Figure S28. HMBC spectrum of racemic LAU. (in $CDCl_3$, 298 K, 400 MHz).

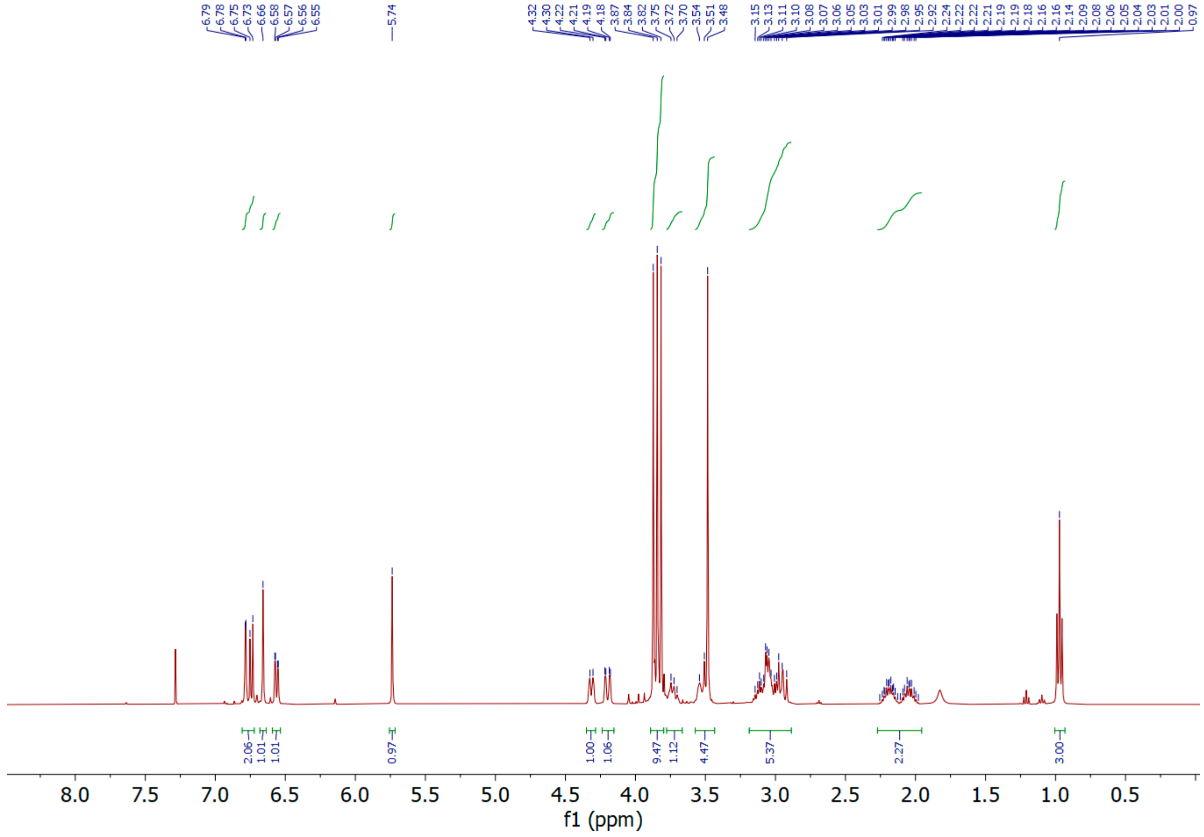

Figure S29. ^{1}H spectrum of racemic PROP. (in $CDCl_3$, 298 K, 400 MHz).

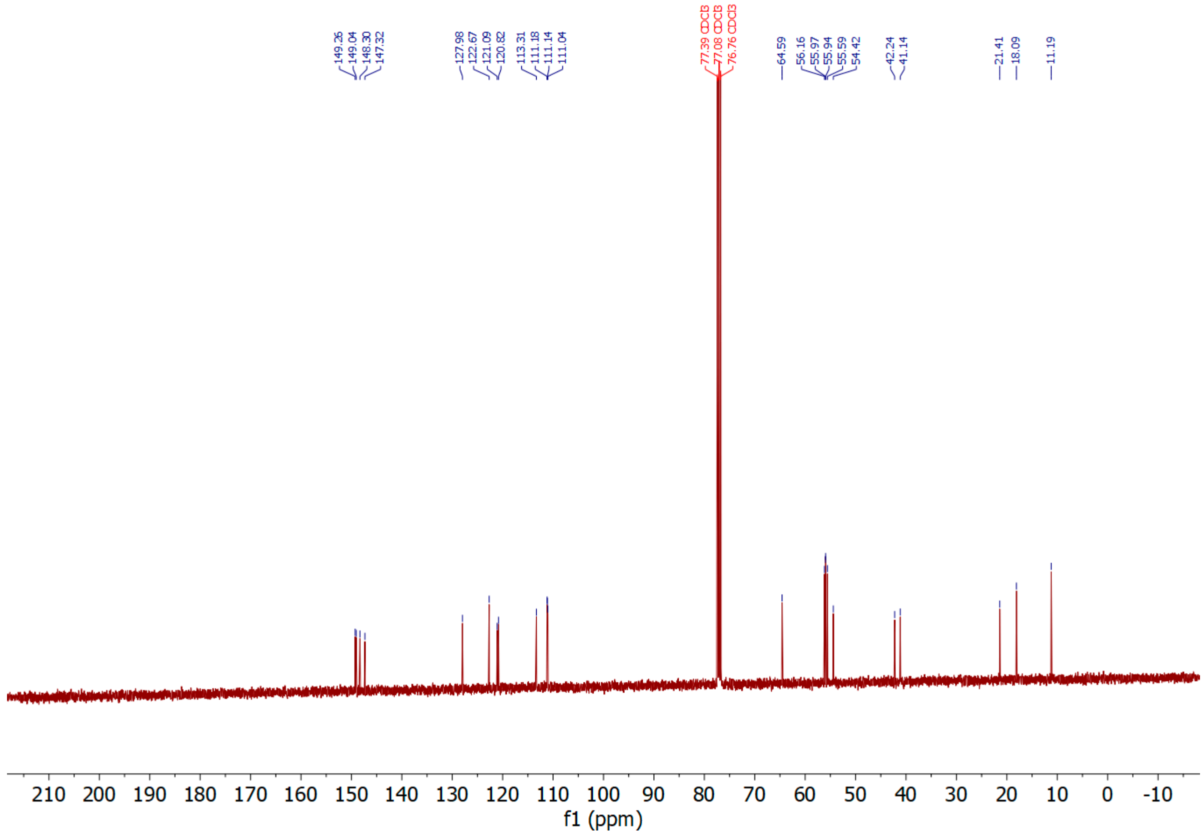

Figure S30. ^{13}C spectrum of racemic PROP. (in $CDCl_3$, 298 K, 400 MHz).

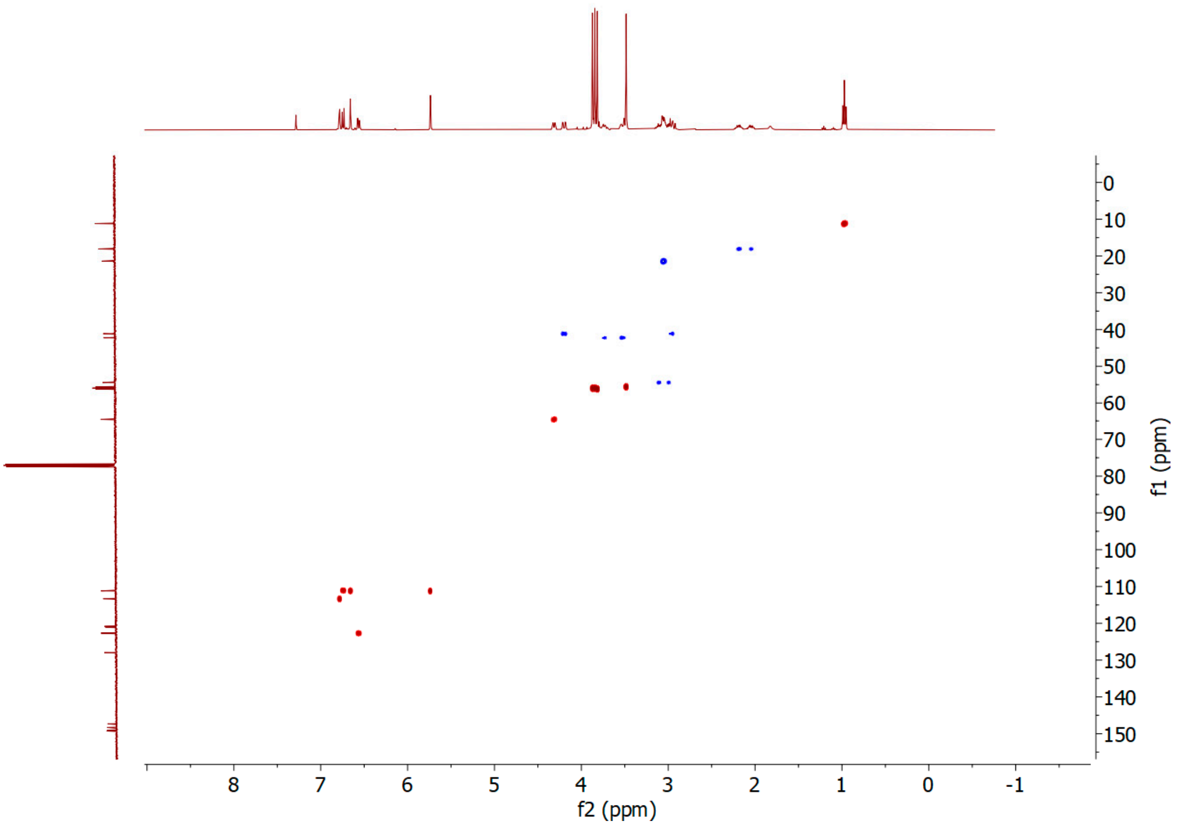

Figure S31. HSQC spectrum of racemic PROP. (in $CDCl_3$, 298 K, 400 MHz).

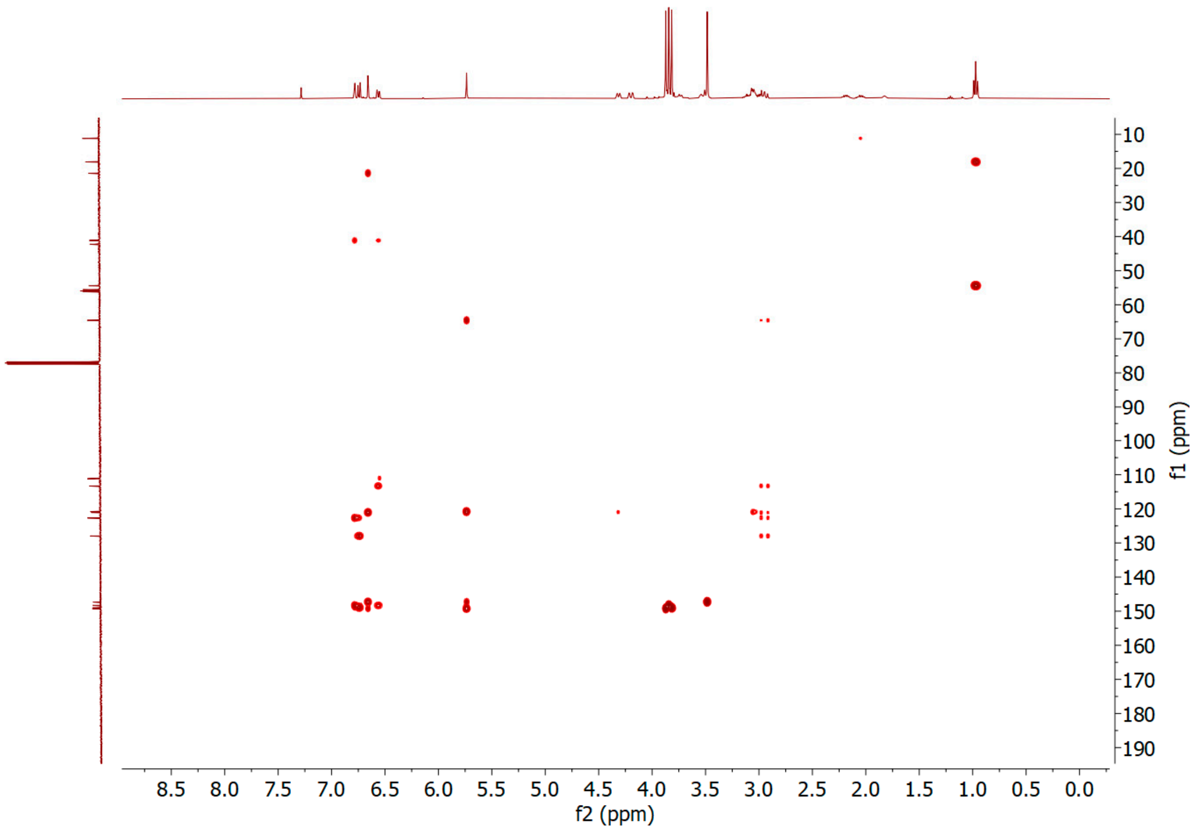

Figure S32. HMBC spectrum of racemic PROP. (in $CDCl_3$, 298 K, 400 MHz).

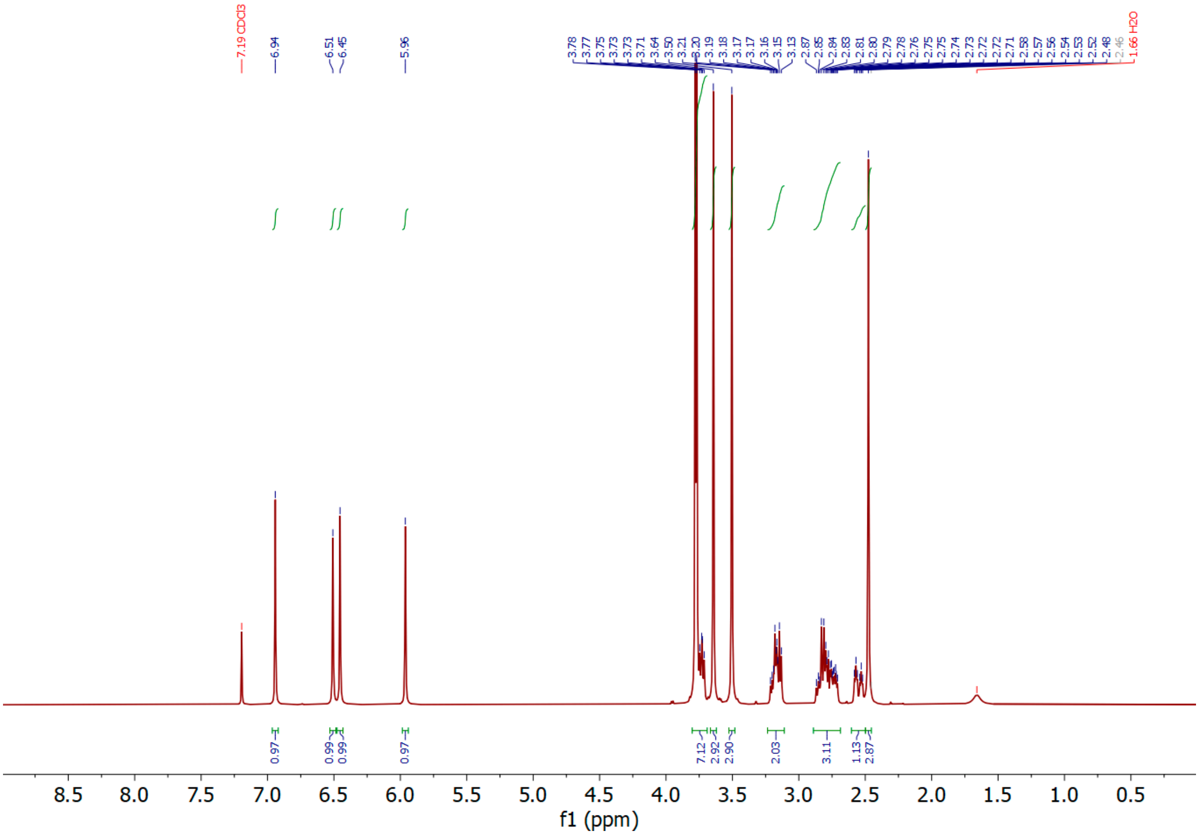

Figure S33. ^{1}H NMR spectrum of racemic Br-LAU. (in $CDCl_3$, 298 K, 400 MHz).

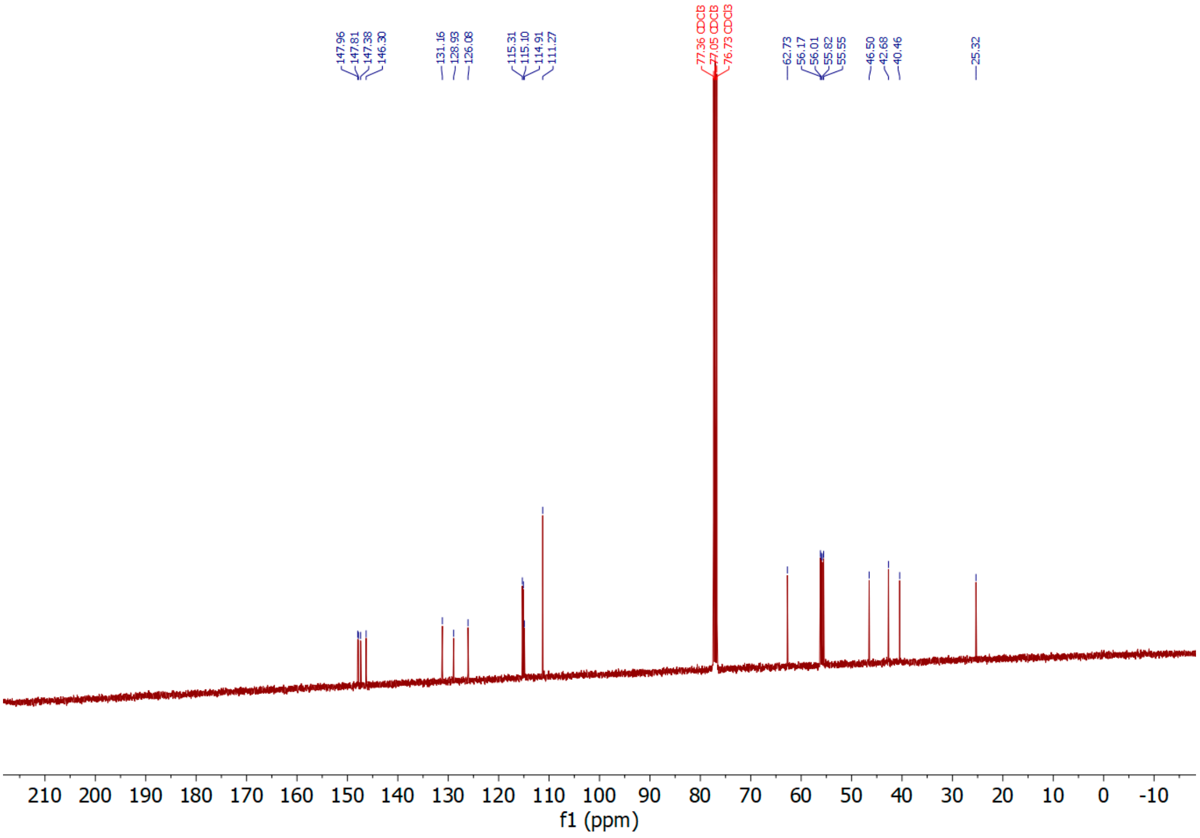

Figure S34. ^{13}C NMR spectrum of racemic Br-LAU. (in $CDCl_3$, 298 K, 400 MHz).

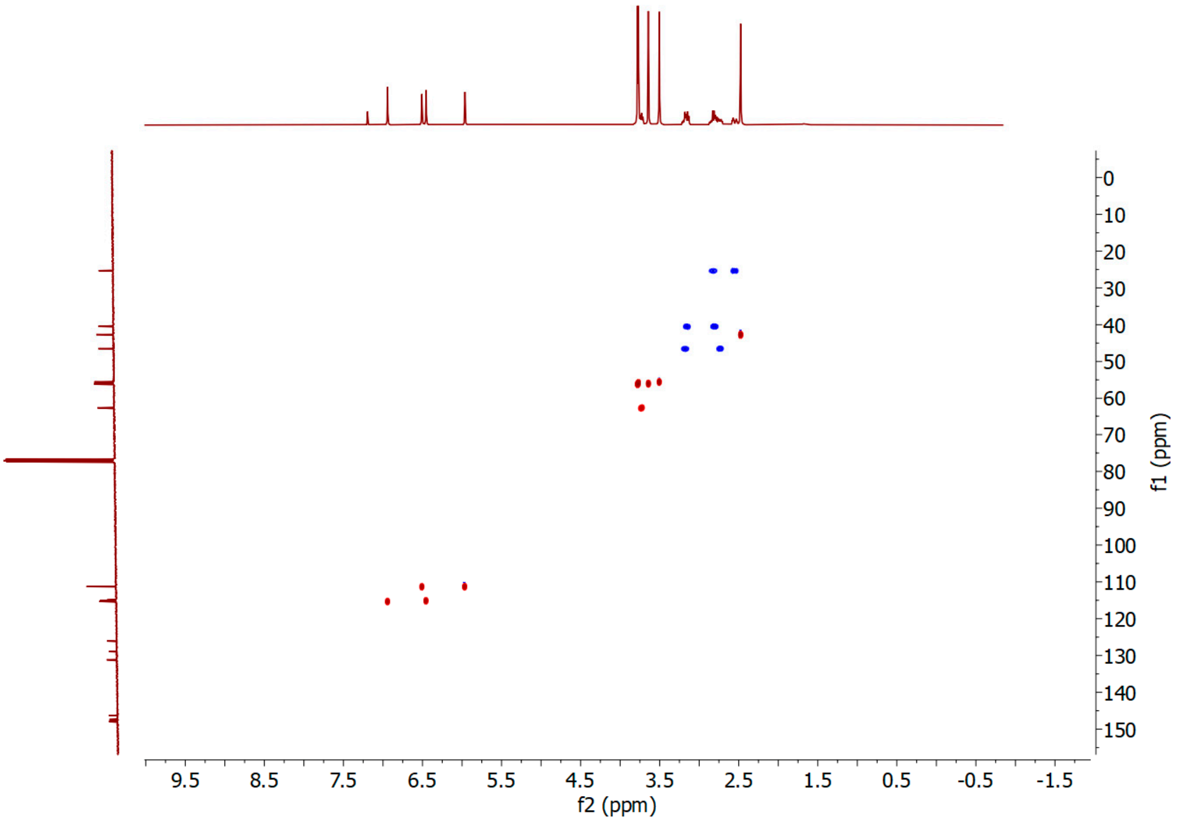

Figure S35. HSQC spectrum of racemic Br-LAU. (in $CDCl_3$, 298 K, 400 MHz).

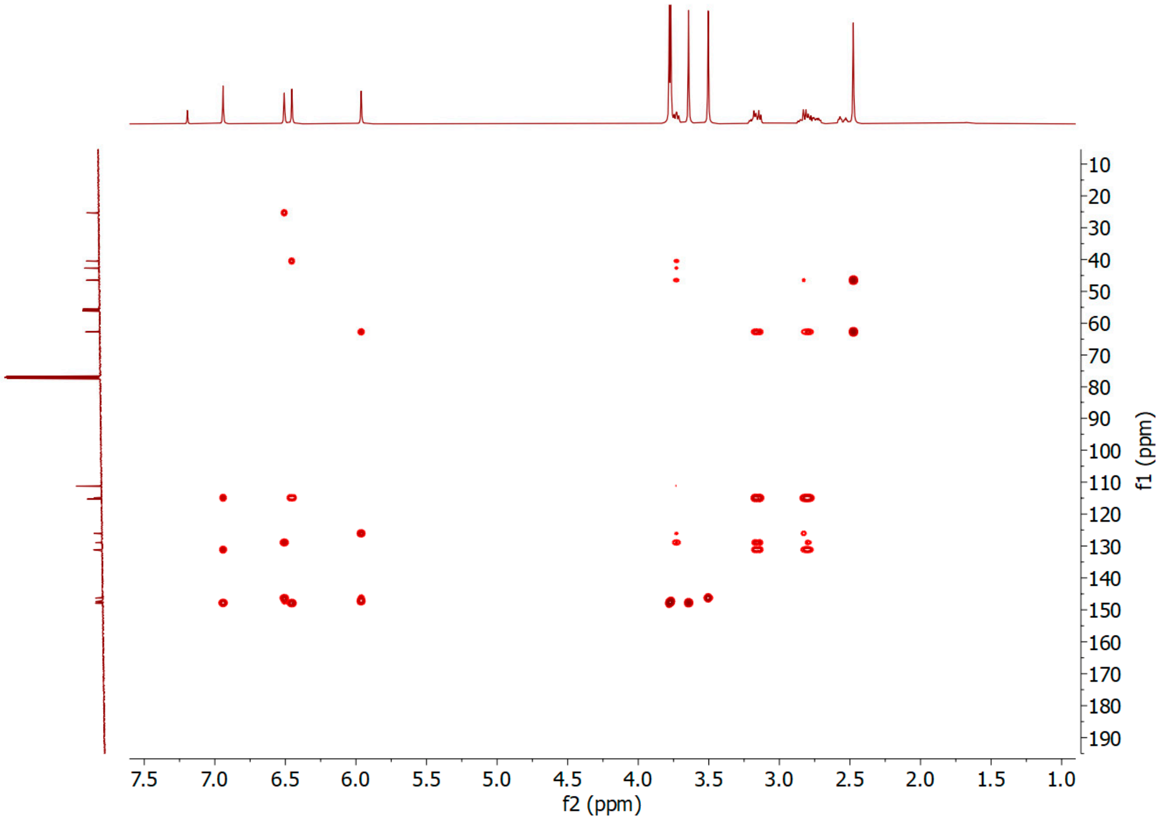

Figure S36. HMBC spectrum of racemic Br-LAU. (in $CDCl_3$, 298 K, 400 MHz).
